# *Streptomyces* polyketides mediate bacteria–fungi interactions across soil environments

**DOI:** 10.1038/s41564-023-01382-2

**Published:** 2023-06-15

**Authors:** Mario K. C. Krespach, Maria C. Stroe, Tina Netzker, Maira Rosin, Lukas M. Zehner, Anna J. Komor, Johanna M. Beilmann, Thomas Krüger, Kirstin Scherlach, Olaf Kniemeyer, Volker Schroeckh, Christian Hertweck, Axel A. Brakhage

**Affiliations:** 1 Department of Molecular and Applied Microbiology, Leibniz Institute for Natural Product Research and Infection Biology (Leibniz-HKI), Jena, Germany; 2 Institute of Microbiology, Friedrich Schiller University Jena, Jena, Germany; 3 Department of Biomolecular Chemistry, Leibniz Institute for Natural Product Research and Infection Biology (Leibniz-HKI), Jena, Germany; 4 Present Address: Department of Microbiology, Karlsruhe Institute of Technology (KIT), Karlsruhe, Germany; 5 Present Address: Leibniz Institute on Aging–Fritz Lipmann Institute (FLI), Jena, Germany

**Keywords:** Applied microbiology, Bacterial physiology

## Abstract

Although the interaction between prokaryotic and eukaryotic microorganisms is crucial for the functioning of ecosystems, information about the processes driving microbial interactions within communities remains scarce. Here we show that arginine-derived polyketides (arginoketides) produced by *Streptomyces* species mediate cross-kingdom microbial interactions with fungi of the genera *Aspergillus* and *Penicillium*, and trigger the production of natural products. Arginoketides can be cyclic or linear, and a prominent example is azalomycin F produced by *Streptomyces iranensis*, which induces the cryptic orsellinic acid gene cluster in *Aspergillus nidulans*. Bacteria that synthesize arginoketides and fungi that decode and respond to this signal were co-isolated from the same soil sample. Genome analyses and a literature search indicate that arginoketide producers are found worldwide. Because, in addition to their direct impact, arginoketides induce a secondary wave of fungal natural products, they probably contribute to the wider structure and functioning of entire soil microbial communities.

## Abstract (web summary)

The soil bacterium *Streptomyces iranensis* triggers the production of natural products in the fungus *Aspergillus nidulans* using arginine-derived polyketide signalling molecules.

## Main

In all known habitats on Earth microorganisms form diverse consortia with a multitude of prokaryotic and eukaryotic microorganisms^1^. These microbial consortia provide services crucial for life^2^. For example, soil systems host a large diversity of microorganisms that contribute to its vital functions such as the regulation of nutrient cycling, decomposition of organic matter, generation of soil structure, suppression of plant diseases and support of plant productivity^3–5^. In particular, the interplay between bacteria and fungi seems to be critical for community functionality, and alteration of the balance between these microorganisms emerges as a potential cause of disease^6,7^. For example, lichens are composed of fungi and phototrophic microorganisms like algae or cyanobacteria^8^. They provide microhabitats for many bacteria, thus forming a complex microbial consortium^9^. Similarly, it was demonstrated that microorganisms from different kingdoms drive the assembly of microbiota in preterm infants^10^. Therefore, elucidation of functional interactions between bacteria and fungi that determine the composition of healthy microbial consortia has attracted increased attention.

We are beginning to understand that microorganisms communicate with and influence each other via a chemical language composed of low-molecular-weight organic compounds that are part of the greater chemical category commonly referred to as natural products (NPs)^11–13^. Some of these compounds have been assigned functions with reference to their impact on humans, that is, as antibiotics or toxins^1,14,15^. However, the ecological role for most of these compounds remains obscure. An important clue towards elucidating this role is the finding that their production can be triggered by surrounding microorganisms^12,16,17^. Consequently, many of the gene clusters in microorganisms encoding the biosynthesis of such NPs are silent under conventional laboratory conditions^18^. Their biosynthesis can only be activated when the correct stimulus is provided, which in many cases is another microorganism^16,19,20^. We reported an early example of such an activation when we showed that *Streptomyces rapamycinicus* and its closest relative *Streptomyces iranensis* specifically trigger the production of orsellinic acid and its derivatives by inducing the transcription of the orsellinic acid (*ors*) biosynthetic gene cluster (BGC) in the fungus *Aspergillus nidulans*^19,21^. The versatility of *S. rapamycinicus* was impressively underlined by the finding that the bacterium also triggers the activation of the *fcc* and *fgn* gene clusters of *Aspergillus fumigatus*, resulting in the production of fumicyclines and fumigermin, respectively^22,23^. However, the bacterial agent triggering the fungal production of NPs remained elusive.

**Extended Data Fig. 1:**
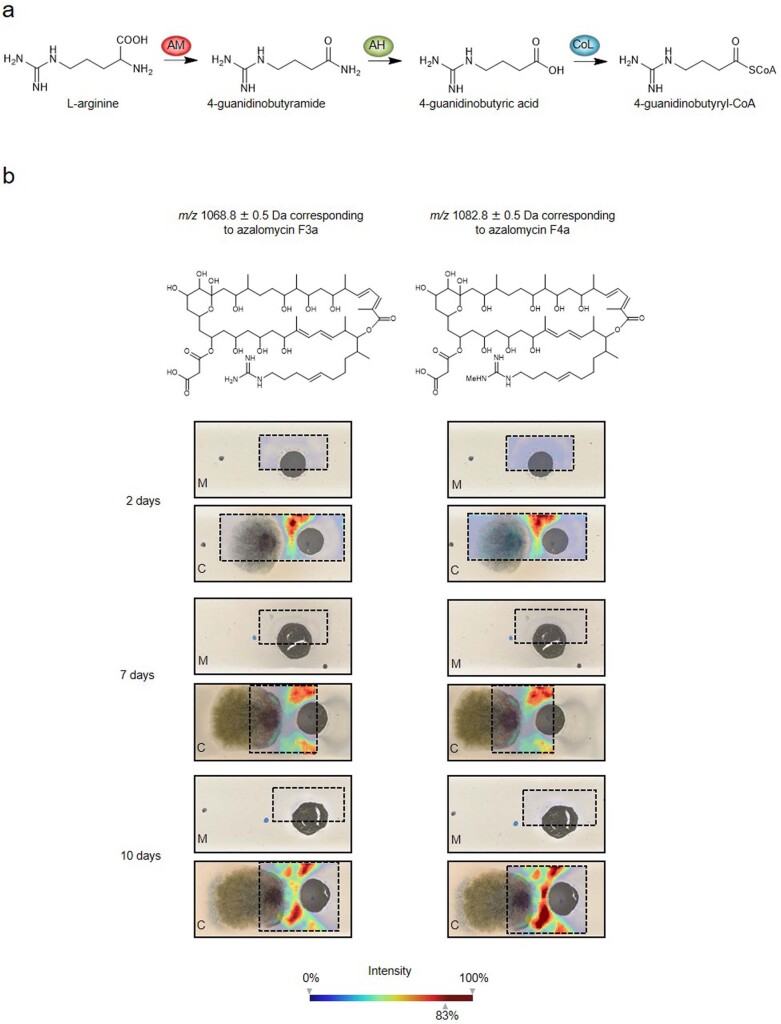
Proposed pathway from arginine to the starter unit of arginoketides and MALDI-IMS time course of co-cultivation of *A. nidulans* with *S. iranensis*. **a**, Proposed pathway from arginine to the starter unit of the arginoketides azalomycin F and clethramycin from *S. violaceusniger*. Figure adapted with permission from ref. ^28^, Wiley. The starter unit 4-guanidinobutyryl-CoA is transferred to the acyl carrier protein domain of the modular polyketide synthases to form arginoketides^28^. AM, arginine monooxygenase; AH, 4-guanidinobutyramide hydrolase; CoL, 4-guanidinobutanoate:CoA ligase. **b**, Visualization of masses corresponding to the main azalomycin F derivatives azalomycin F3a (*m*/*z* 1,068.8 ± 0.5) and azalomycin F4a (*m*/*z* 1,082.8 ± 0.5) produced by *S. iranensis* (right on the glass slide) after 2, 7 and 10 d of co-cultivation with *A. nidulans* (left on the glass slide). Abundances of the analysed masses are depicted as a heat map from low abundance (blue) to high abundance (red) within the measured part (indicated by dashed lines). M, monoculture *S. iranensis*; C, co-culture of *S. iranensis* with *A. nidulans*.

Here we report the discovery of the stimulus—that is, the arginine-derived polyketides that we refer to as arginoketides. These compounds trigger the biosynthesis of NPs in various fungi. All studies so far indicate that the common feature of arginoketides is the origin of their starter unit from arginine. This amino acid undergoes a series of transformations including truncation before the remaining building block of 4-guanidinobutyrate is loaded onto the polyketide synthase^24^ (Extended Data Fig. 1a).

Based on their chemical structure, arginoketides can be divided into two groups: linear and cyclic arginoketides. Members of the cyclic group were previously named marginolactones^25^ and can be further divided into two sub-groups: guanidyl-marginolactones are characterized by a guanidyl moiety in their side chain and include azalomycin F^26^, and amino-marginolactones such as desertomycin A and monazomycin contain a terminal amino group formed from an arginine-derived guanidyl moiety by a cluster-encoded agmatinase^24,27^. Similarly, linear arginoketides also start with arginine as a biosynthetic precursor; however, they lack cyclization and remain linear^28^.

Potential ecological effects of arginoketides on microorganisms have been previously reported. For example, cyclic arginoketides were suggested to promote symbioses by shaping a lichen-like association between the soil-isolated green alga *Chlamydomonas reinhardtii* and *A. nidulans*^21^.

Our data suggest that these compounds, whose producers are found worldwide, impact the surrounding microorganisms directly and, by inducing a secondary wave of diverse NPs in fungi, may structure the composition of microbial consortia.

## Results

### *S. iranensis bld* mutants do not induce fungal *ors* genes

**Fig. 1:**
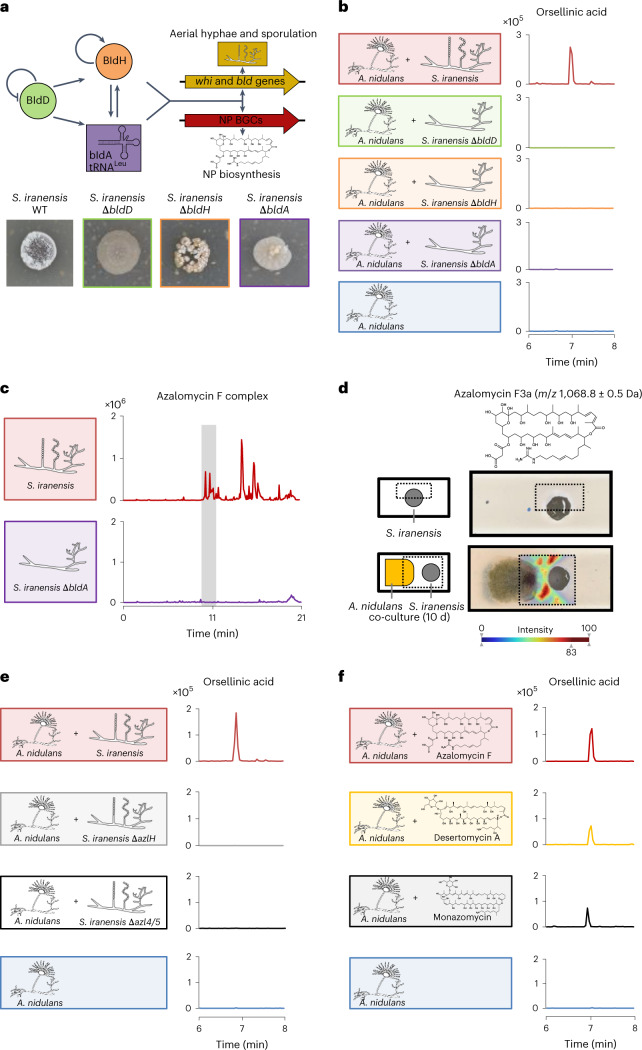
Identification of *Streptomyces*-derived arginoketides as inducing agents for the production of orsellinic acid and its derivatives by the fungus *A. nidulans*. **a**, Regulatory cascade of *bld* genes (top). The Δ*bldD*, Δ*bldH* and Δ*bldA* deletion mutants of *S. iranensis* lack aerial hyphae and spore formation (bottom). **b**, Extracted ion chromatograms for orsellinic acid (*m*/*z* 167 [M-H]^−^) derived from HPLC–MS analysis of the culture supernatants of *A. nidulans* co-cultured with WT and mutant (Δ*bldD*, Δ*bldH* and Δ*bldA*) *S. iranensis* strains. **c**, Total-ion chromatogram of culture extracts of *S. iranensis* WT and Δ*bldA*. The grey box indicates the elution time for the azalomycin F complex found only in the *S. iranensis* WT strain. **d**, MALDI-IMS analysis of *S. iranensis* co-cultured with *A. nidulans* for 10 d on agar. Schematic visualization of the sample preparation (left); the box indicates the measured area. MALDI-IMS analysis of the distribution of azalomycin F3a (*m*/*z* 1,068.8 ± 0.5 Da; right). A heat map was used to depict the abundance of the analysed mass, from low (blue) to high (red) abundance. **e**, Extracted ion chromatogram of orsellinic acid (*m*/*z* 167 [M-H]^−^) derived from HPLC–MS analysis of the co-culture extract of *A. nidulans* with azalomycin F-deficient *S. iranensis* mutants. **f**, Extracted ion chromatograms of orsellinic acid (*m*/*z* 167 [M-H]^−^) derived from HPLC–MS analysis of the culture extracts of *A. nidulans* with the indicated arginoketides. Values depicted on the *y* axes indicate peak intensities.

To identify the bacterial trigger of the silent fungal *ors* BGC, we deleted well-characterized Bld regulators of streptomycetes (Fig. 1a and Supplementary Fig. 1a–c). The designation *bld* for bald originates from *bld* mutants that are characterized by a lack of aerial mycelium^29^. The proteins BldD and BldH as well as the leucine transfer recognising the TTA codon (tRNA^UUA^) encoded by *bldA* are known to regulate streptomycete development and NP biosynthesis^30–33^. Generated *bldD*, *bldA* and *bldH* deletion mutants of *S. iranensis* (Δ*bldD*, Δ*bldH* and Δ*bldA*, respectively) exhibited the expected bald phenotype and interestingly failed to induce the production of orsellinic acid when co-cultured with *A. nidulans* (Fig. 1a,b). Thus, genes controlled by the Bld regulon regulate the bacteria-induced activation of the fungal *ors* BGC.

Proteome, transcriptome and liquid chromatography (LC)–mass spectrometry (MS)-based metabolome data of the Δ*bldA* deletion mutant indicated downregulation of numerous genes and decreased levels of proteins, including members of the azalomycin F BGC, compared with the wild-type (WT) strain (Table 1). This finding is supported by the lack of molecular masses corresponding to azalomycin F in culture extracts of the Δ*bldA* mutant strain compared with the WT (Fig. 1c). In addition, several other BGCs were downregulated in *S. iranensis* Δ*bldA* compared with the WT. These BGCs are listed in Supplementary Table 1.

**Table 1:** Changes in gene expression and relative protein abundance in the *S. iranensis* Δ*bldA* mutant compared with the WT

Gene name	Gene ID	mRNA steady-state level (log_2_(Δ*bldA*/WT fold change))	Protein abundance (log_2_(Δ*bldA*/WT fold change)
*azl5*	SIRAN1022	−6,91	−1,54
*azl4*	SIRAN1023	−7,28	−4,58
*azlA*	SIRAN1024	−7,54	−3,69
*azlH*	SIRAN1025	−7,52	−4,43
*azlG1*	SIRAN1026	−7,32	−6,88
*azlG2*	SIRAN1027		−4,34
*azlF*	SIRAN1028		−5,04
*azlE1*	SIRAN1029	−7,60	−2,90
*azlE2*	SIRAN1030		−4,51
*azlE3*	SIRAN1031		−3,16
*azlD1*	SIRAN1032		−3,04
*azlD2*	SIRAN1034		−2,59
*azlD3*	SIRAN1036		−4,41
*azlD4*	SIRAN1037		−4,69
*azlC1*	SIRAN1038		−2,85
*azlC2*	SIRAN1039		−2,73
*azlB1*	SIRAN1040		−2,88
*azlB2*	SIRAN1042		−4,00
*azlB3*	SIRAN1043		−3,60

Steady-state mRNA levels and relative protein abundance of core biosynthetic genes/proteins of the azalomycin F BGC. Data are depicted as the log_2_-transformed fold change of the transcripts-per-kilobase-million values and protein abundance measured in the Δ*bldA* mutant compared with the WT. No value for the log_2_-transformed fold change for the mRNA steady-state level indicates that no detectable transcripts mapped to these genes.

### *S. iranensis* secretes azalomycin F in the presence of *A. nidulans*

To visualize the spatial distribution of azalomycin F, we applied matrix-assisted laser desorption-ionization (MALDI) imaging MS (IMS) to co-cultures of *S. iranensis* and *A. nidulans*. Two metabolites likely to be representing azalomycin F3a and azalomycin F4a^34^ were detected in the interaction zone (Fig. 1d and Extended Data Fig. 1b), which agreed well with the calculated mass for azalomycin F3 with [M + H]^+^ 1,068.6583. Their distribution suggests that azalomycin biosynthesis is activated in the cells proximal to the fungus. After 10 d of co-culture (Fig. 1d and Extended Data Fig. 1b), azalomycin F co-localized with the fungus, which agrees with our HPLC–MS data indicating that azalomycin F accumulates in the biomass fraction of *A. nidulans* (Supplementary Fig. 2). This finding suggests that azalomycin F is produced to affect *A. nidulans* under the conditions applied and underlines the affinity of the compound to the fungal biomass.

### Azalomycin F triggers NP biosynthesis in *A. nidulans*

**Extended Data Fig. 2:**
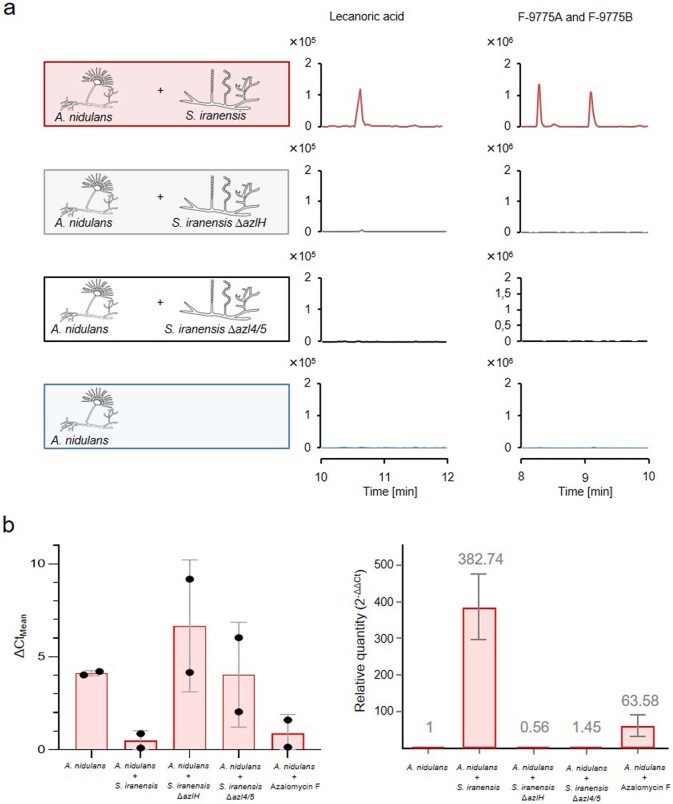
Analysis of orsellinic acid derivatives and expression of orsellinic acid biosynthesis gene *orsA* of *A. nidulans* during co-cultivation of the fungus with *S. iranensis* or addition of azalomycin F to the fungal culture. **a**, Co-culture of *A. nidulans* with *S. iranensis* WT and mutant strains Δ*azlH* and Δ*azl4*Δ*azl5*, and extracted ion chromatogram of lecanoric acid (*m*/*z* 317 [M-H]^−^) and the isobaric compounds F-9775A and F-9775B (*m*/*z* 395 [M-H]^−^) derived from HPLC–MS analysis of culture supernatant. **b**, RT–qPCR analysis of the orsellinic acid biosynthesis gene *orsA* of *A. nidulans* co-cultured with the indicated *S. iranensis* strains or in monoculture supplemented with azalomycin F. The numbers indicate the fold increase of transcripts. Left: Δ*C*t_Mean_ values of two independent experiments, each in 5–6 biological replicates. Error bars display the s.d. Right: Numbers indicate fold increase of transcripts with error bars showing maximal and minimal relative quantity, which was calculated using the QuantStudio Design and Analysis software. Source data

**Extended Data Fig. 3:**
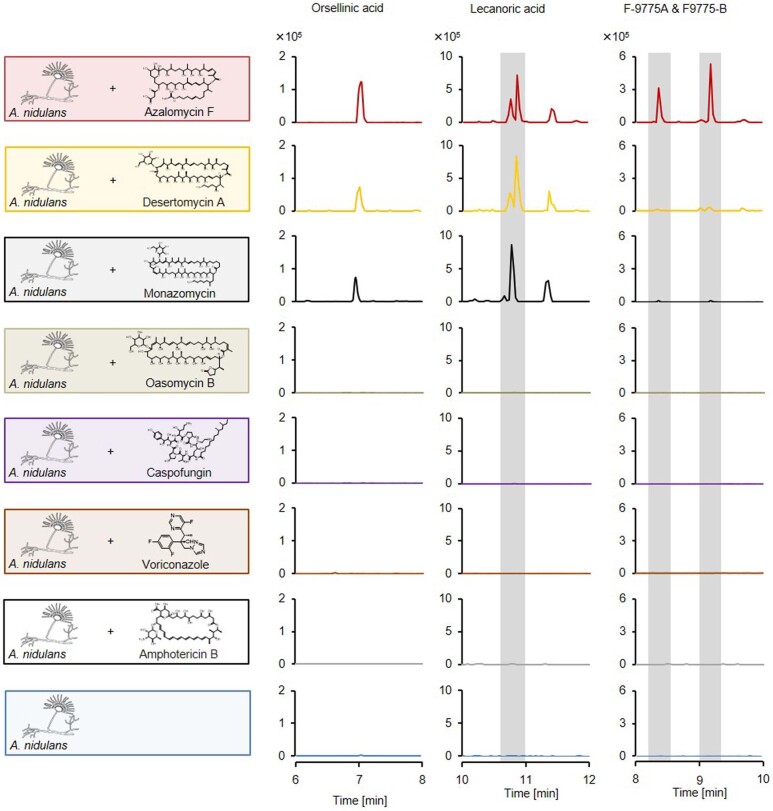
Analysis of cyclic arginoketides, their derivatives as well as membrane- and cell-wall-disturbing compounds for their *ors* BGC-inducing activity. Cultivation of *A. nidulans* supplemented with indicated compounds and extracted ion chromatograms for orsellinic acid (*m*/*z* 167 [M-H]^−^), lecanoric acid (*m*/*z* 317 [M-H]^−^), and F-9775A and F-9775B (*m*/*z* 395 [M-H]^−^) derived from HPLC–MS analysis of culture extracts.

**Extended Data Fig. 4:**
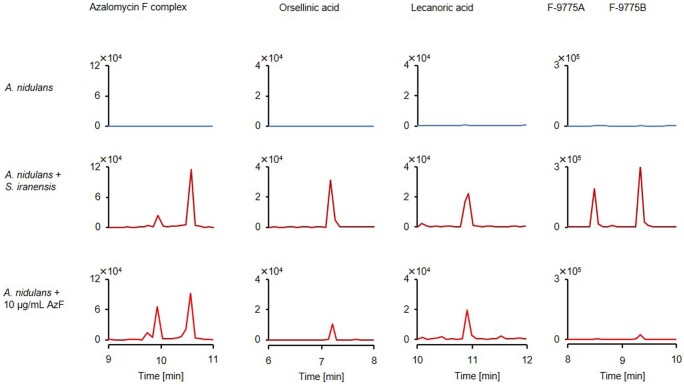
Azalomycin F at 10 µg ml^−1^ is in the same concentration range as azalomycin F produced by *S. iranensis* in co-culture with *A. nidulans*. Cultivation of *A. nidulans* in monoculture, co-culture with *S. iranensis* or supplemented with 10 µg ml^−1^ azalomycin F. Extracted ion chromatograms for azalomycin F3a (*m*/*z* 1069 [M + H]^+^), orsellinic acid (*m*/*z* 167 [M-H]^−^), lecanoric acid (*m*/*z* 317 [M-H]^−^), and F-9775A and F-9775B (*m*/*z* 395 [M-H]^−^) derived from HPLC–MS analysis of culture extracts.

To demonstrate that azalomycin F is a bacterial signal triggering fungal NP biosynthesis, we analysed mutant strains with deleted azalomycin F-biosynthesis genes^21,28,35^. Co-cultivation of the respective *S. iranensis* mutant strains Δ*azlH* and Δ*azl4*Δ*azl5* with *A. nidulans* did not result in the production of orsellinic acid or its derivatives (Fig. 1e and Extended Data Fig. 2a). As an important proof we added 10 µg ml^−1^ purified azalomycin F to monocultures of *A. nidulans* and observed induced production of orsellinic acid, lecanoric acid as well as F-9775A and F-9775B (Fig. 1f and Extended Data Fig. 3). This azalomycin F concentration is comparable to that of co-cultures of *A. nidulans* and *S. iranensis* (Extended Data Fig. 4). The production of fungal compounds was accompanied by increased levels of steady-state messenger RNA of the orsellinic acid biosynthesis gene *orsA* (Extended Data Fig. 2b). Collectively, azalomycin F is the sought-after bacterial trigger that activates NP biosynthesis in *A. nidulans*. To rule out that the response is specific for the *A. nidulans* strain investigated, we added an equal concentration of azalomycin F to another WT *A. nidulans* strain named FGSC A4. This strain also reacted to azalomycin F with the production of orsellinic acid, lecanoric acid as well as the compounds F-9775A and F-9775B (Supplementary Fig. 3).

### Arginoketides induce fungal NP biosynthesis

**Extended Data Fig. 5:**
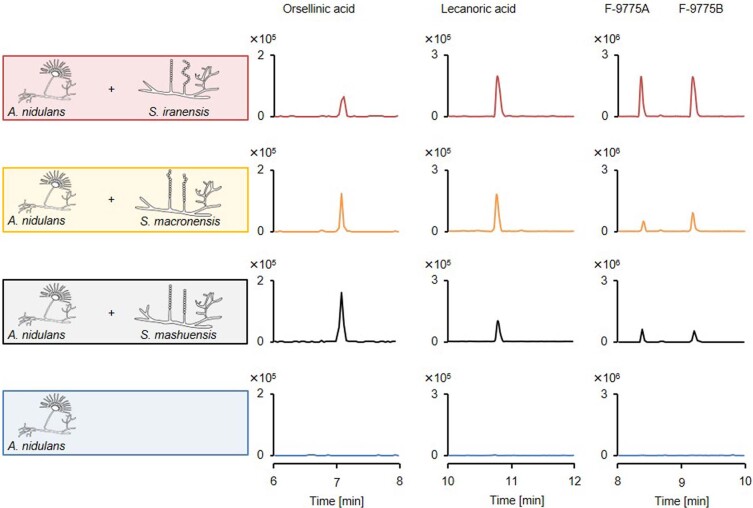
Production of *ors* compounds by *A. nidulans* triggered by arginoketide-producing streptomycetes. Co-cultures of *A. nidulans* with the indicated *Streptomyces* species and extracted ion chromatograms for orsellinic acid (*m*/*z* 167 [M-H]^−^), lecanoric acid (*m*/*z* 317 [M-H]^−^), and F-9775A and F-9775B (*m*/*z* 395 [M-H]^−^) derived from HPLC–MS analysis of culture supernatant.

Cyclic arginoketides like azalomycin F consist of a macrolactone backbone and a side chain containing a guanidyl or amino group^25^. Further cyclic arginoketides such as desertomycin A and monazomycin are produced by *Streptomyces macronensis* and *Streptomyces mashuensis*, respectively^27,36^. Given that we found that purified desertomycin A and monazomycin also induced the production of orsellinic acid and its derivatives in *A. nidulans* (Fig. 1f and Extended Data Fig. 3), we proposed that actinomycetes producing these compounds are able to induce the fungal *ors* gene cluster. This is indeed the case, as co-cultures of *S. macronensis* or *S. mashuensis* with *A. nidulans* contained orsellinic acid and its derivatives (Extended Data Fig. 5).

We also tested whether oasomycin B^37^, a desertomycin-family compound lacking the amino group in its side chain, induces fungal NP biosynthesis. The importance of the amino moiety is reflected by the observation that oasomycin B had lost the antibacterial activity assigned to desertomycin A^38^ and the reconstitution of a positively charged moiety to oasomycin restores antibacterial activity^39^. In comparison to desertomycin A, equimolar concentrations of oasomycin B only minimally triggered the production of orsellinic acid and its derivatives by the fungus (Extended Data Fig. 3). Thus, we concluded that the positively charged moieties of the desertomycins may be required for the induction of fungal NP biosynthesis. This could be true for all cyclic arginoketides; however, this needs to be proven for each compound separately.

To determine the specificity of the inducing activity of arginoketides, we tested known antifungal compounds that either act on cell-wall biosynthesis, like caspofungin, or on the fungal membrane, such as amphotericin B or voriconazole^40^. In contrast to arginoketides, none of the structurally different antimycotics, at least at the concentrations tested, induced production of the compound (Extended Data Fig. 3). The same was found for rapamycin, a prominent NP of *S. iranensis*, which also did not induce fungal NP biosynthesis (Supplementary Fig. 4).

### Worldwide distribution of arginoketide-producing bacteria

**Fig. 2:**
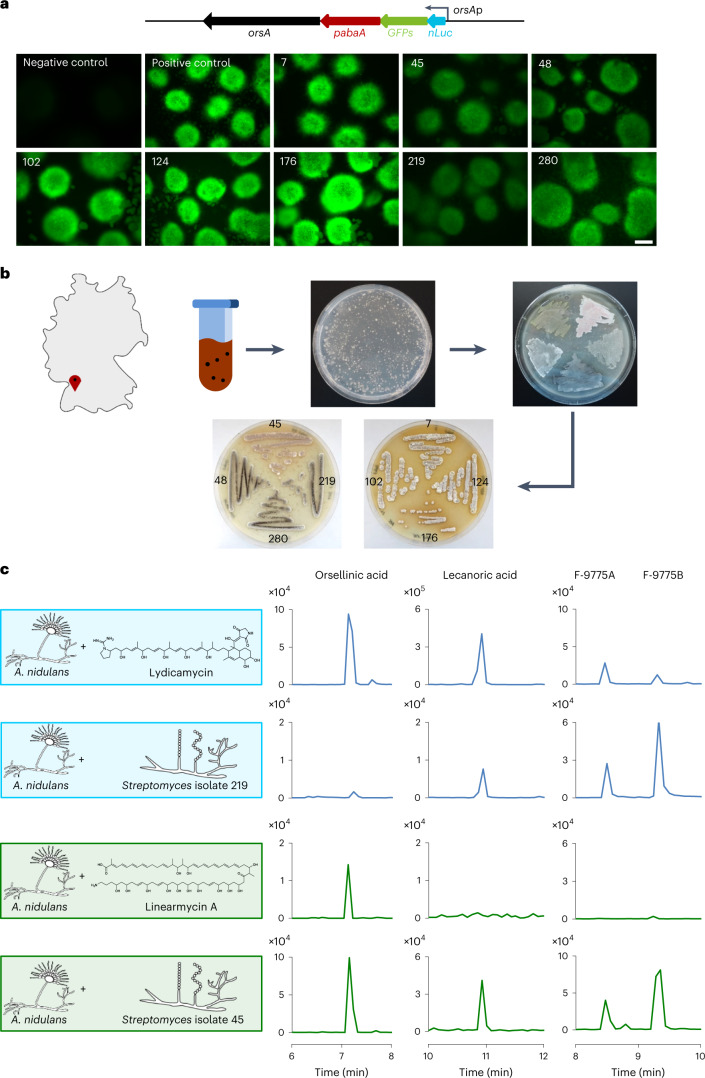
Isolation of soil bacteria producing arginoketides and inducing the *ors* BGC of *A. nidulans*. **a**, Schematic depiction of the *orsA*p-*nLuc*-*GFPs* reporter gene fusion (top) and images of the *A. nidulans orsA*p-*nLuc*-*GFPs* reporter strain co-cultured with *Streptomyces* soil isolates 7, 45, 48, 102, 124, 176, 219 and 280 for 6 h (bottom). The negative control was a *A. nidulans orsA*p-*nLuc*-*GFPs* monoculture and the positive control was *A. nidulans orsA*p-*nLuc*-*GFPs* co-cultured with the known inducer *S. iranensis*. Scale bar, 500 µm. Images are representative of at least three independent co-cultures. **b**, Map of Germany with the soil sample origin indicated (top left) and workflow for the isolation of bacteria (right). **c**, Cultivation of *A. nidulans* with lydicamycin, linearmycin and soil isolates *Streptomyces* isolates 219 and 45. Extracted ion chromatograms of orsellinic acid and its derivatives from HPLC–MS analyses of the culture extracts are shown (right). Ion chromatograms for orsellinic acid (*m*/*z* 167 [M-H]^−^), lecanoric acid (*m*/*z* 317 [M-H]^−^) as well as F-9775A and F-9775B (*m*/*z* 395 [M-H]^−^) derived from HPLC–MS analysis of culture extracts. Values depicted on the *y* axes indicate peak intensities.

**Fig. 3:**
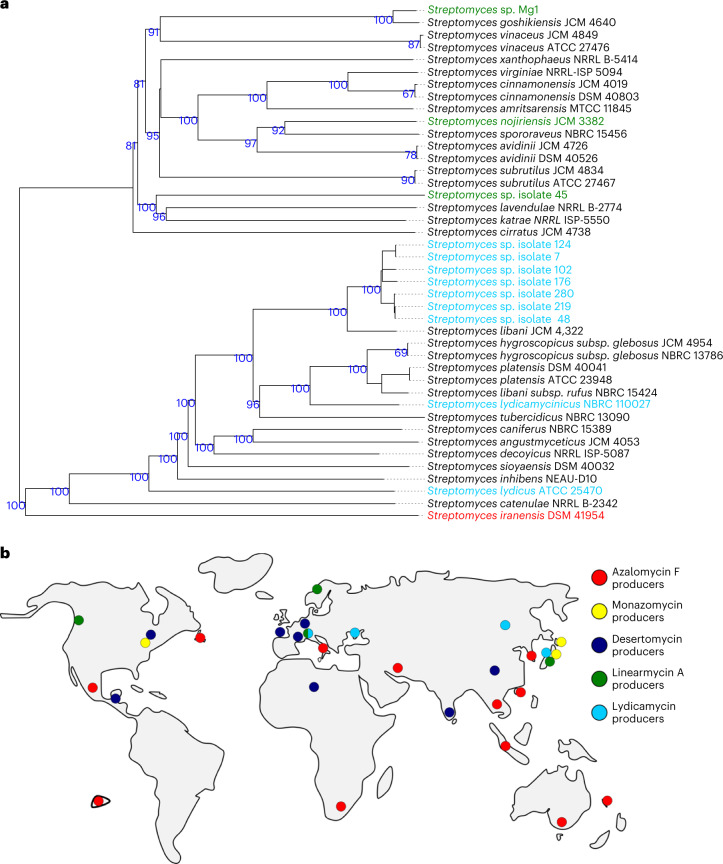
Phylogenetic tree of arginoketide producers, with a focus on strains isolated here, and the worldwide occurrence of arginoketide producers. **a**, Genomes of soil isolates producing lydicamycin and linearmycin were compared with known producers of these compounds and *S. iranensis* using the Type Strain Genome Server provided by the DSMZ^63^. Isolates 7, 48, 102, 124, 176, 219 and 280 (blue) are closely related to each other. According to the ANI values, they represent strains of *S. libani*. Isolate 45 (green) differs from the other isolates. Green, linearmycin A producers; light blue, lydicamycin producers; red, azalomycin F-producing *S. iranensis*. The genomes of the isolated streptomycetes are accessible under NCBI BioProject PRJNA830323. **b**, World map without Antarctica indicating where bacteria producing the indicated arginoketides were isolated based on genome and literature analyses.

**Extended Data Fig. 6:**
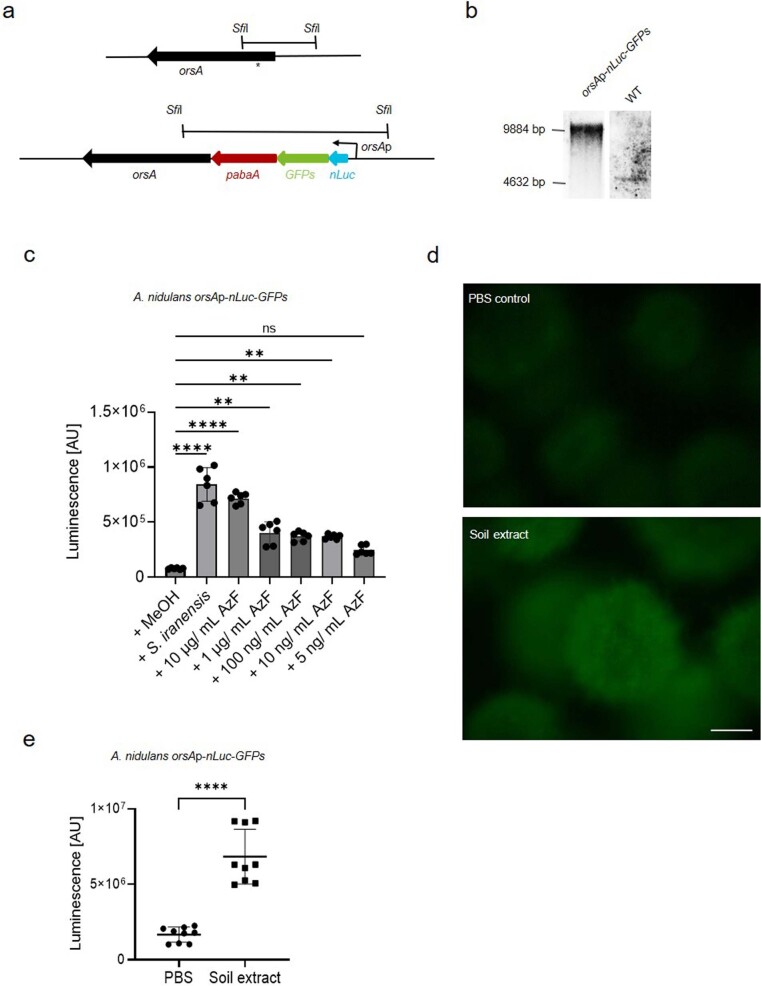
Genomic organization, Southern blot analysis and testing of the *A. nidulans orsA*p-*nLuc*-*GFPs* reporter strain. **a**, Schematic overview of the *orsA* genomic locus in the WT (bottom) and *orsA* genomic locus (top) of the *A. nidulans* reporter strain containing the *orsA* promoter 5′ of the *nLuc*-*GFPs* translational gene fusion. The probe used for Southern blot analysis is indicated by an asterisk. **b**, Southern blot analysis of genomic DNA of the WT and *orsA*p-*nLuc*-*GFPs* reporter strain. Genomic DNA was digested with SfiI overnight. The probe (*) was directed against the upstream region of the *orsA* gene (see **a**). The WT strain gives a band of 4,632 bp, whereas for the reporter strain a band of 9,884 bp is characteristic of the integrated gene fusion at the *orsA* locus. **c**, Nanoluciferase-derived luminescence of *A. nidulans orsA*p-*nLuc*-*GFPs* reporter strain after the addition of *S. iranensis*, azalomycin F (AzF) or methanol (MeOH) as control. *n* = 6 biological replicates examined over three experiments. Error bars represent the mean ± s.e.m. Each treatment was compared with the methanol control using Dunnett’s multiple comparison within an ordinary one-way ANOVA test. ****P ≤ 0.0001; **P ≤ 0.01 (1 µg ml^−1^ AzF, *P* = 0.0015; 100 ng ml^−1^, *P* = 0.0054; 10 ng ml^−1^, *P* = 0.0059); NS, *P* = 0.2757. Concentrations as low as 10 ng ml^−1^ AzF were reliably detected. **d**, Fluorescence images of *A. nidulans orsA*p-*nLuc*-*GFPs* reporter strain incubated for 20 h with PBS (PBS control) or soil extract. Scale bar, 500 µm. **e**, Nanoluciferase-derived luminescence of *A. nidulans orsA*p-*nLuc*-*GFPs* reporter strain after the addition of PBS (control) or a PBS extract of soil used in this study for the isolation of fungi and filamentous bacteria. *n* = 9 biological replicates over three experiments. Error bars represent the mean ± s.e.m. ***P < 0.0001 (unpaired, two-tailed *t*-test). Source data

To facilitate and accelerate testing of compounds for *ors* BGC activation, we generated a *GFPs*-*orsA* reporter strain that fluoresces green following *orsA* activation (Fig. 2a and Extended Data Fig. 6a–d). To determine the frequency at which arginoketide producers occur in nature, we then isolated actinomycetes from a soil sample collected from a previously unsampled location near Eutingen im Gäu, Germany (48.4661535°N, 8.7291989°E). From 600 mg of soil we were able to culture 305 filamentous bacteria representing potential actinomycetes (Fig. 2b). When the isolated strains were co-cultured with the *A. nidulans orsA*p*-nLuc-GFPs* reporter strain, eight of them triggered fluorescence (Fig. 2a). The genomes of these bacterial strains were sequenced by Illumina sequencing. Using the German Collection of Microorganisms and Cell Cultures (Deutsche Sammlung von Mikroorganismen und Zellkulturen, DSMZ) Type Strain Genome Server, we investigated the phylogeny of our isolates. Isolates 7, 48, 102, 124, 176, 219 and 280 are either closely related to each other or the same species (Fig. 3a). According to the calculated average nucleotide identity (ANI) values (Extended Data Table 1) they most probably represent isolates of *Streptomyces libani*. Isolate 45, however, is clearly different from the other isolates, as confirmed by the ANI value. Therefore, we continued working with isolates 45 and 219, the latter as a representative for strains 7, 48, 102, 124, 176, 219 and 280.

Using antiSMASH and BlastN, the obtained genomes for isolate 45 and 219 were analysed for the unusual arginine-loading domain in polyketide synthases required for the biosynthesis of arginoketides. The genome of bacterial isolate 219 bears a potential lydicamycin BGC (Supplementary Fig. 5a and Supplementary Table 2), whereas isolate 45 carries a putative BGC for linearmycins (Supplementary Fig. 5b and Supplementary Table 3). This finding is interesting because it extends the spectrum of inducing compounds to linear arginoketides. Subsequent high-performance LC–MS (HPLC–MS), high-resolution MS (HR-MS) and tandem MS (MS/MS) analyses of the culture extracts of bacterial isolates 219 and 45 confirmed the production of 30-demethyllydicamycin and a linearmycin-family compound (Supplementary Fig. 6a,b). This was supported by the finding that commercially available linearmycin A and 30-demethyllydicamycin induced the formation of orsellinic acid and its derivatives when added to cultures of *A. nidulans* (Fig. 2c). We hypothesized that the producing strains should also be capable of inducing the fungal compounds. This was indeed the case, as formation of orsellinic acid and its derivatives was triggered by the *Streptomyces* isolates 219 and 45 producing lydicamycin and linearmycin, respectively (Fig. 2c). Therefore, not only cyclic but also linear arginoketides are able to induce fungal NP biosynthesis. A genome and literature analysis of arginoketide-producing bacteria demonstrated their worldwide distribution (Fig. 3b and Extended Data Table 2). Thus, it is very probable that there are far more species and strains producing arginoketides.

It is challenging to identify NPs in the soil using analytical techniques because of their tendency to adsorb to soil particles^41^. Therefore, we evaluated the sensitivity of our reporter strain towards azalomycin F and found that it responded to concentrations as low as 10 ng ml^−1^ (Extended Data Fig. 6c). This motivated us to add soil supernatant to a culture of the *A. nidulans* reporter strain, which led to a clear increase in activity of the nLuc compared with a culture without soil supernatant (Extended Data Fig. 6d,e). Because our results so far indicated that the *ors* cluster is specifically induced by arginoketides, these data suggest that arginoketides are indeed present in soil.

### Frequency of fungal responders to arginoketides

**Extended Data Fig. 7:**
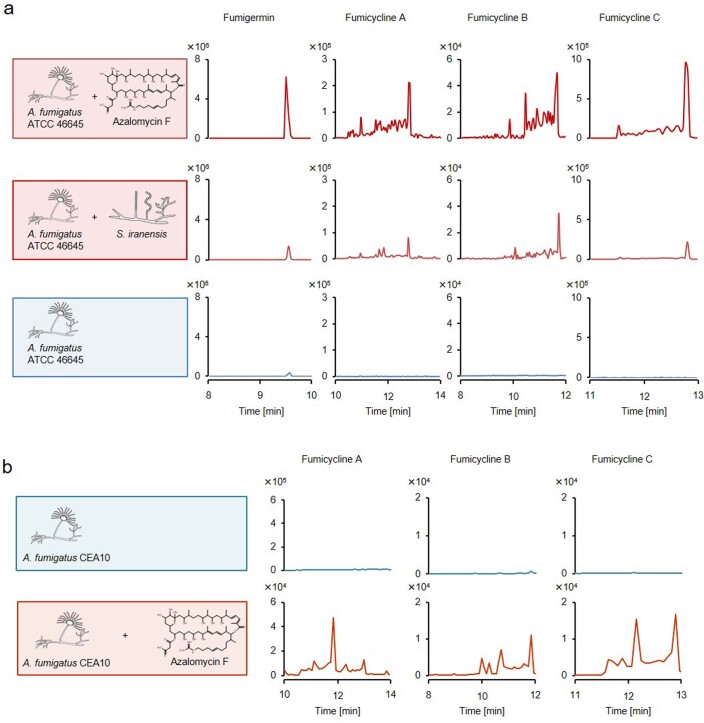
Production of fumigermin and fumicyclines A–C in *A. fumigatus* triggered by azalomycin F. **a**, Cultures of *A. fumigatus* ATCC 46645 were supplemented with azalomycin F or, as a control, co-incubated with *S. iranensis*. Extracted ion chromatograms for fumigermin (*m*/*z* 195 [M + H]^+^), fumicycline A (*m*/*z* 423 [M-H]^−^), fumicycline B (*m*/*z* 441 [M-H]^−^) and fumicycline C (*m*/*z* 483 [M-H]^−^) derived from HPLC–MS analysis of culture extracts are shown. **b**, Cultures of *A. fumigatus* CEA10 were supplemented with azalomycin F. Extracted ion chromatograms for fumicycline A (*m*/*z* 423 [M-H]^−^), fumicycline B (*m*/*z* 441 [M-H]^−^) and fumicycline C (*m*/*z* 483 [M-H]^−^) derived from HPLC–MS analysis of culture extracts are shown. *A. fumigatus* CEA10 only carries a non-functional fumigermin BGC and thus this compound was not produced^22^.

To obtain insights into whether arginoketides are sensed by fungi other than *A. nidulans*, we analysed *A*. *fumigatus*, a human pathogen that causes life-threatening infections. Despite being phylogenetically distantly related to *A. nidulans*, *A. fumigatus*^42^ also specifically reacts to *S. rapamycinicus* and *S. iranensis* through the activation of the BGCs for fumicyclines and fumigermin^22,23^. When we added purified azalomycin F to monocultures of the clinical isolate *A. fumigatus* ATCC 46645, it produced fumicyclines as well as fumigermin. Similarly, the clinical isolate *A. fumigatus* CEA10 produced fumicyclines when treated with azalomycin F, indicating that these fungi also respond to azalomycin F (Extended Data Fig. 7a,b).

**Fig. 4:**
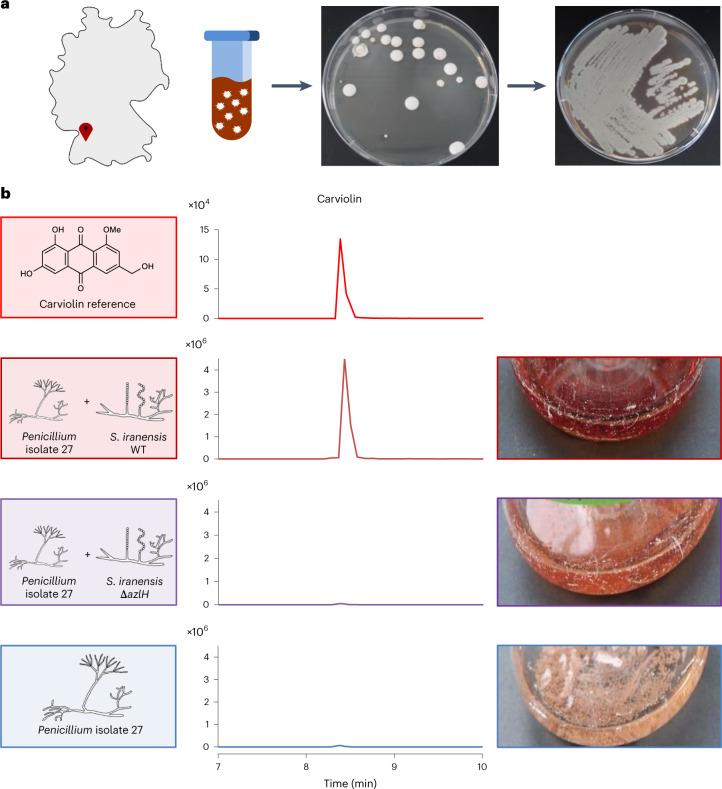
Isolation of *Penicillium* isolate 27 and identification of its response to azalomycin F by the production of the red pigment carviolin. **a**, Map of Germany with the origin of the soil sample indicated (left) and the workflow for the isolation of fungi (right). **b**, Extracted ion chromatograms for carviolin (*m*/*z* 299 [M-H]^−^) derived from HPLC–MS analysis of the culture extracts following mono- and co-culture of *Penicillium* isolate 27 with *S. iranensis* WT and the azalomycin F-deficient mutant strain *S. iranensis* Δ*azlH* (middle). A carviolin reference was included (top). Images of the cultures are provided (right). Values depicted on the *y* axes indicate peak intensities.

Motivated by this finding, we sought to determine the frequency at which potential signal-responsive fungi occur. For this purpose, we isolated filamentous fungi from the same soil sample used for the isolation of filamentous bacteria (Fig. 4a). We isolated a total 106 fungal strains and tested their response to *S. iranensis* WT and the *S. iranensis* Δ*azlH* mutant strain. Of the 106 fungal strains, 31 showed a change in culture coloration when co-cultured with *S. iranensis* WT that was not seen for the Δ*azlH* deletion mutant (Supplementary Fig. 7a). Furthermore, we performed HPLC–MS analyses of these (co-)cultures (Supplementary Fig. 8). Full ion chromatograms of four representative (co-)cultures showed new mass peaks only when the isolated fungi were co-cultured with the *S. iranensis* WT, suggesting induced production of NPs. We were able to identify the fungal compound whose production was triggered by *S. iranensis* for one strain. Analyses of HPLC–MS, HR-MS and MS/MS spectra indicated that this fungal isolate produces carviolin in the presence of *S. iranensis* WT but not in the presence of the Δ*azlH* mutant strain (Fig. 4b and Supplementary Fig. 6c). Carviolin is a red pigment of *Penicillium* species^43^ with potential silkworm-attracting properties^44^. In agreement with this finding, fungal isolate 27 also overproduced carviolin in the presence of the linearmycin-producing bacterial isolate 45 and 30-demethyllydicamycin-producing bacterial isolate 219 (Supplementary Fig. 7b).

**Extended Data Fig. 8:**
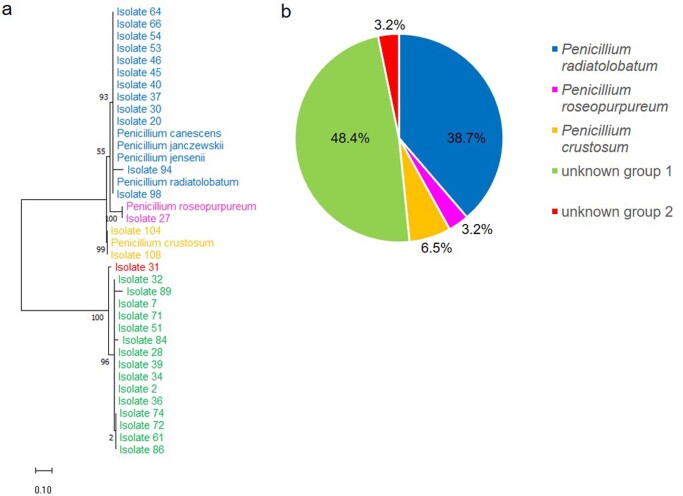
Phylogenetic tree of fungal isolates responding to arginoketides. **a**, Phylogenetic tree of fungal isolates based on MEGA X. The maximum likelihood method and the Jukes–Cantor model were used. Bootstrap values obtained after 500 replications are indicated at the nodes. The tree is drawn to scale, with branch length measured in the number of base substitutions per site. Blue, *P. radiatolobatum* group; magenta, *P. roseopurpureum* group; yellow, *P. crustosum* group; green, unknown group 1; red, unknown group 2. **b**, Frequency of isolation of fungal species and unknown species from soil. Groups 1 and 2 are defined as ‘unknown’. Based on their ITS sequences, they did not group with each other and with a type strain from the NCBI database. ITS sequences are accessible at Genbank under the accession numbers ON307333–ON307363.

Sequencing of the internal transcribed spacer (ITS) regions 1 and 2 suggested that all tested fungal soil isolates belong to the genus *Penicillium* (Extended Data Fig. 8a,b). This finding expands the group of signal-responsive fungi by the genus *Penicillium*, which is well known for its capability to produce NPs^45^. Collectively, our data indicate the presence of widespread microbial consortia wherein bacteria biosynthesize arginoketides and fungi decode this molecular signal.

## Discussion

The identification of arginoketides as bacterial inducers of fungal NP biosynthesis was based on a comprehensive analysis of the *bld* regulatory system in *S. iranensis*. This system had been previously shown to regulate both the development—that is, processes leading to the production—of spores and biosynthesis of a number of NPs in other streptomycetes^29–33^. Here we extend these findings to a function of *bld* genes for triggering cross-kingdom interactions. A number of NP biosyntheses have been previously linked to the *bld* system. For example, deletion of *bldD* encoding a developmental regulator in *Streptomyces ghanaensis* completely abolished the biosynthesis of the antibiotic moenomycin among other NPs such as desferrioxamine B and oxohygrolidin^46,47^. At this stage it can only be speculated which ecological advantage the bacteria have from a connection between sporulation and activation of fungal NP BGCs. It has been shown that unfavourable conditions for the streptomycete trigger sporulation^17,30^. The simultaneous stimulation of fungi to produce NPs might help protect the habitat against invaders when the bacteria are less protected.

**Fig. 5:**
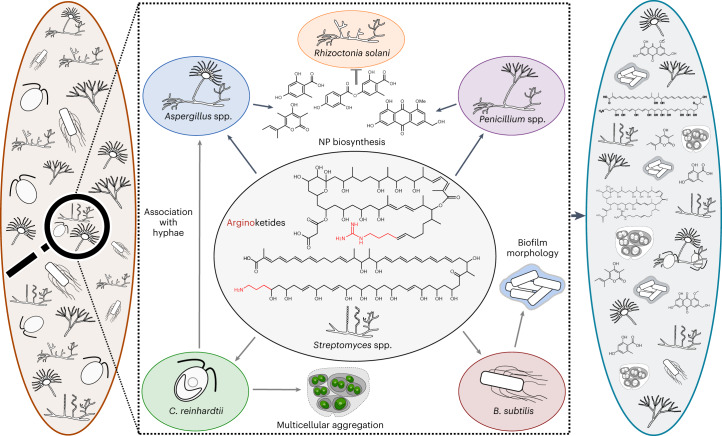
Model summarizing potential versatile structural changes to a microbial consortium enacted by arginoketides and the fungal NPs produced in response. A microbial consortium (left) that consists of stochastically assembled microorganisms without the influence of arginoketides. Proven effects mediated by the indicated arginoketides here (black arrows) and by others (grey arrows) are shown (middle square). The production of arginoketides may thus lead to a differently structured microbial consortium (right). Some *Streptomyces* species produce arginoketides whose release is most probably triggered by *A. nidulans* and *C. reinhardtii*^21^ (middle). Arginoketides, whose producers are found worldwide, can impact surrounding microorganisms by inducing the formation of multicellular aggregates (gloeocapsoids) in *C. reinhardtii*^34^ and a distinct biofilm by *B. subtilis*^52,53^. Furthermore, they trigger the association of the green alga with *A. nidulans*^21^, and they trigger and enhance the biosynthesis of fungal NPs that themselves have the potential to affect surrounding microorganisms^51^ that are either included in or excluded from a consortium. Details on the specific arginoketides mediating the indicated effects are provided in the main text.

Arginoketides are low-molecular-weight compounds produced by distantly related actinomycetes. As shown here they seem to share the conserved ability to induce the production of NPs in phylogenetically diverse fungi, at least in co-cultures in the laboratory (Figs. 2,4, Extended Data Figs. 7,8 and Supplementary Figs. 7,8). Therefore, it is tempting to hypothesize that they have a major impact on their surrounding microorganisms (Fig. 5); they directly impact surrounding microorganisms by inducing the production of fungal NPs that themselves influence other microorganisms.

As shown here *A. nidulans* responds to sublethal concentrations of arginoketides with the production of orsellinic and lecanoric acid, which were shown to be produced by lichens^48^. It is worth noting that the release of azalomycin F by *S. iranensis* increases in the presence of *A. nidulans* (Fig. 1d and Extended Data Fig. 1b). Whether this bacterial response is triggered by a signal by the fungus or induction due to starvation caused by exploitation of nutrients by *A. nidulans* remains to be determined. However, a potential effect of cyclic arginoketides is to promote symbioses—for example, the compounds shape a lichen-like association between the soil-isolated green alga *C. reinhardtii* and *A. nidulans* that might have contributed to the evolution of lichens consisting of fungi and algae^34^. At sublethal concentrations the arginoketide azalomycin F leads to the formation of a novel multicellular structure named gloeocapsoid that confers some protection to algal cells^34^ and, most interestingly here, azalomycin F triggers green algae to accumulate and thereby hide in fungal mycelia from the adverse effects of azalomycin F^21^ (Fig. 5). Moreover, these compounds also have the potential to impact the spatial partitioning of microorganisms in a microbial consortium. In accordance with this, azalomycin F as well as desertomycin A were described to induce transition of hyphal growth into yeast-like morphology in the dimorphic fungus *Paecilomyces viridis*^49^, which probably changes their potential to explore new habitats. In addition, desertomycin A was shown to induce premature phialide formation and sporulation of this fungus^50^.

Interestingly, the induced *A. nidulans* compound lecanoric acid has been found to specifically inhibit the growth of the plant-pathogenic basidiomycete *Rhizoctonia solani*^51^. It is thus conceivable that by triggering the production of NPs in the isolated fungi, arginoketides have a far-reaching effect on which microorganisms are included in and excluded from a microbial consortium (Fig. 5).

It is worth noting that not only fungi and green algae respond to arginoketides; these compounds also have signalling function to bacteria. Linearmycin A activates a two-component system in *Bacillus subtilis*, resulting in the induction of a specific biofilm morphology^52,53^. Together, arginoketides might have been involved in the evolution of multicellularity, shaping a lichen-like association between *C. reinhardtii* and *A. nidulans* that might have contributed to the evolution of lichens, induces NP biosynthesis in fungi and serves as signals to other bacteria. This underlines the influence of these compounds on numerous neighbouring microorganisms^34^.

Based on their biosynthetic origin and function we propose that arginoketides form a group of compounds sharing structural similarities and function. Their arginine-derived amino or guanidyl moiety seems to be essential for the activity of these compounds, given that oasomycin B without such a group lost its inducing activity (Extended Data Fig. 3) and had no antibacterial activity, unless a positively charged moiety was reintroduced to the molecule^39^. However, not only the biosynthesis of these compounds is distinct. Their function also seems to be specific given that other antifungal compounds at the concentrations tested here did not induce NP production in *A. nidulans*. As some cyclic arginoketides have been shown to interact with the membrane^21,54^, it was conceivable that their effect is connected to membrane damage or antifungal activity. However, the membrane-damaging antifungal compounds amphotericin B and voriconazole as well as compounds disturbing the fungal cell-wall-like caspofungin did not exhibit inducing activity at the concentrations tested here, highlighting the specificity of arginoketides and that simple antifungal activity is not a trigger for the induction of the fungal NP biosynthesis analysed here.

Here we have shown that arginoketides serve as targeted signal molecules produced by bacteria and perceived by fungi. Furthermore, we suggest that this is a universal signalling system, evidenced by the straightforward co-isolated bacteria–fungal pairs and the fact that producing bacteria can be found on virtually all continents. The producing bacteria represent phylogenetically diverse streptomycetes (Fig. 3a), suggesting that production of this signal molecule is advantageous for numerous different bacteria. The presence of arginoketides in soil is also supported by the observation that our sensor strain responded to soil supernatant, although it cannot be excluded that also other compounds induce the sensor strain. The receiver fungi thus far identified belong to the genera *Penicillium* and *Aspergillus*, genera that are widespread in nature, including soil. These findings indicate a widespread phylogenetic as well as global distribution of this type of communication. Furthermore, the ubiquity of arginoketide producers is not limited to soil—*Streptomyces althioticus* producing desertomycin G was isolated from the surface of a seaweed in the Cantabrian Sea^55^.

In summary, the wide distribution of actinomycetes producing these compounds on virtually all continents and the ease with which fungi decoding this chemical signal are isolated from soil suggest that arginoketides represent a universal component of the microbial communication network shaping microbial communities.

## Methods

### Microorganisms, plasmids, media and cultivation

All microbial strains and plasmids used in this study are listed in Supplementary Table 4. The primers used are listed in Supplementary Table 5.

### Cultivation of microorganisms

Wild-type and deletion mutants of *S. iranensis* DSM41954 (HM35^T^) as well as *Streptomyces macronensis* UC 8271 (NRRL12566) and *Streptomyces mashuensis* DSM40896 were cultured as described by Krespach and colleagues^34^. *Streptomyces* species soil isolates 7, 45, 48, 102, 124, 176, 219 and 280 were inoculated (2.5 × 10^7^ spores) in tryptic soy broth with yeast extract (TSBY)^35^ in Erlenmeyer flasks with cottonwool plugs and incubated at 28 °C with shaking (180 r.p.m.) for 3 d. To generate spores, 200–300 µl of densely grown cultures were streaked on oatmeal agar plates, which were incubated at 28 °C for 14 d and the spores were harvested.

*A. nidulans* RMS011, *A. nidulans* FGSC A4, *A. fumigatus* ATCC 46645 and *A. fumigatus* CEA10 were cultured in *Aspergillus* minimal medium (AMM)^56^. For *A. nidulans*, the AMM was supplemented with 1 ml l^−1^ trace elements (22 g l^−1^ FeSO_4_·7H_2_O, 5 g l^−1^ ZnSO_4_·7H_2_O, 1.6 g l^−1^ CuSO_4_·5H_2_O, 5 g l^−1^ MnSO_4_·H_2_O, 11 g l^−1^ Na_2_B_4_O_7_·7H_2_O, 1.1 g l^−1^ (NH_4_)_6_Mo_7_O_24_·4H_2_O) and 0.3 mM FeSO_4_. For *A*. *nidulans* RMS011, the AMM was additionally supplemented with 5 mM l-arginine and 3 µg ml^−1^ *p*-amino benzoic acid (PABA)^57^. For *A. fumigatus*, the AMM was supplemented with Hutner’s trace elements^58^. For all strains, 3 × 10^8^ spores were inoculated into 50 ml AMM and incubated at 37 °C with shaking at 200 r.p.m. to generate precultures.

### Co-culture of *Aspergillus* species with *Streptomyces* species

Four-day-old precultures of *Streptomyces* species were set up as described in the previous section. Mycelia of overnight cultures of *A. fumigatus* or *A. nidulans* (approximately 16 h old) in AMM medium were separated from the medium using Miracloth (Merck Millipore) and placed in fresh AMM (*A. nidulans* was supplemented with 1 ml l^−1^ trace elements, 0.3 mM FeSO_4_ and, when needed to complement auxotrophies, 3 µg ml^−1^ PABA and 5 mM l-arginine)^57^. A 1/20 of the final culture volume from the streptomycete culture^19^, 2–40 µg ml^−1^ purified arginoketide—that is, desertomycin A (30 µg ml^−1^), monazomycin A (40 µg ml^−1^), azalomycin F (10 µg ml^−1^), linearmycin A (2 µg ml^−1^), lydicamycin (20 µg ml^−1^) or oasomycin B (30 µg ml^−1^)—or 0.6–2.56 µg ml^−1^ antifungal compound—that is, caspofungin (0.6 µg ml^−1^), voriconazole (2 µg ml^−1^) or amphotericin B (2.56 µg ml^−1^)—were added to the culture, followed by further incubation at 37 °C with shaking at 200 r.p.m. Desertomycin A, lydicamycin and monazomycin were purchased from Santa Cruz Biotechnology, and linearmycin A and oasomycin B from BioAustralis. Azalomycin F was purified from *S. iranensis* as described in the ‘Production and purification of azalomycin F from *S. iranensis*’ section. Samples for HPLC–MS analyses were taken after 12 h (for *A. fumigatus*) and 24 h (for *A. nidulans*) of co-cultivation and extracted as described in the following section.

### Isolation of filamentous bacteria from soil

Surface soil was collected near the castle ruin ‘Eutinger Tal’ in Eutingen im Gäu, Baden-Württemberg, Germany (48.4661535°N, 8.7291989°E) on 24 June 2021. This site was chosen due to a lack of agricultural use and forestry as a consequence of its status as a protected landscape. To the best of our knowledge, this site has not been subject to isolation of microorganisms before and hence no sampling bias was predicted. A total of approximately 600 mg of soil was used to isolate filamentous bacteria. On three separate occasions, about 200 mg of soil was mixed with 3 ml PBS and thoroughly vortexed. The samples were allowed to sediment for 1.5–2 h, after which the supernatants were transferred to new tubes and heated for 10 min at 50 °C to induce spore germination. The samples were allowed to cool for 10 min at room temperature. Undiluted samples and samples diluted 1:100 in PBS were streaked on TAP-agar^59^ with 100 µg ml^−1^ cycloheximide and 50 µg ml^−1^ nalidixic acid to inhibit the growth of fungi and Gram-negative bacteria. The agar plates were subsequently incubated at 28 °C for 7–14 d. Single colonies that had a fuzzy appearance were re-streaked on oatmeal agar. After these microorganisms had re-grown, colonies were inoculated into 3 ml glucose, yeast and malt (GYM), 3 ml tryptic soy broth (TSB), and 3 ml M79 media^60^. As soon as the cultures had grown to a high density, they were co-cultured with the *A. nidulans* *orsA*p-*nLuc*-*GFPs* strain to evaluate their ability to activate the *ors* BGC. Green-fluorescent protein spark (GFPs)-inducing bacteria were co-cultured with *A. nidulans* RMS011 and the production of orsellinic acid and its derivatives was evaluated by HPLC–MS. Bacteria inducing fungal orsellinic acid production were subjected to genome sequencing using an Illumina NextSeq 2000 system (paired-end sequencing, read length of 150 nucleotides, 100× coverage, 10 × 10^6^ reads; StarSeq). Genomic DNA was isolated using a NucleoSpin microbial DNA mini kit (Macherey–Nagel) according to the manufacturer’s manual. Potential NP BGCs were identified using antiSMASH^61^ and the BLAST algorithm^62^. Whole-genome-based phylogenetic analysis was carried out using the Type Strain Genome Server provided by the DSMZ^63^. Genomic data of the isolated *Streptomyces* strains are accessible under NCBI BioProject PRJNA830323. Genome sequences of the other *Streptomyces* strains were obtained from the NCBI genomes database (https://www.ncbi.nlm.nih.gov/genome/).

### Isolation of filamentous fungi from soil

Filamentous fungi were isolated from the same soil sample used for the isolation of bacteria. The soil (200 mg) was mixed with 3 ml PBS and thoroughly vortexed. The sample was allowed to sediment for 1.5–2 h. Neat samples and samples diluted 1:10 and 1:100 were plated on malt extract agar plates (20 g l^−1^ malt extract, 2 g l^−1^ yeast extract, 10 g l^−1^ glucose, 0.25 g l^−1^ NH_4_Cl, 0.25 g l^−1^ K_2_HPO_4_ and 20 g l^−1^ agar, pH 6.0) supplemented with 50 µg ml^−1^ nalidixic acid and 25 µg ml^−1^ kanamycin to inhibit the growth of bacteria. The agar plates were incubated at 28 °C for 3–17 d. Single fungal colonies were re-streaked on AMM agar containing Hutner’s trace elements^56,58^. Spores were harvested with 5 ml 0.9% (wt/vol) NaCl and stored at −80 °C in 50% (vol/vol) glycerol. For screening of their response to azalomycin F, the fungal isolates were co-cultured with *S. iranensis* or 10 µg ml^−1^ azalomycin F in 24-well plates in a Thermo-shaker PST-60HL (Biosan). For this purpose, 1 ml AMM-Hutner’s medium was inoculated with 1–10 × 10^6^ spores and cultured for 2 d at 28 °C with shaking at 600 r.p.m. When fungal growth was observed, the medium was replaced with fresh AMM medium supplemented with Hutner’s trace elements, and 50 µl of an *S. iranensis* culture or 10 µg ml^−1^ azalomycin F were added to the fungal mycelium. The co-culture was incubated for 1–2 d at 28 °C and 600 r.p.m. All isolates showing a visible colour change when co-cultured with *S. iranensis* or azalomycin F compared with the fungus alone were further investigated. Candidate fungal isolates were precultured in 50 ml AMM supplemented with Hutner’s trace elements and co-cultured in 15 ml fresh AMM supplemented with Hutner’s trace elements with the WT or ∆*azlH* mutant strain of *S. iranensis* (750 µl of culture). The formation of NPs was evaluated by HPLC–MS after 1–2 d. To determine the genus of the isolated fungi, their genomic DNA was isolated using the method described by Schroeckh and colleagues^19^. The ITS1 and ITS2 regions flanking the 5.8S ribosomal DNA of the isolates were PCR amplified using proof-reading Phusion high-fidelity DNA polymerase (Thermo Fisher Scientific) and the primers ITS1 and ITS4 (ref. ^64^). Sequencing was carried out by LGC Genomics and the obtained sequences were analysed using the BLAST algorithm and MEGA X^62,65^. The carviolin standard used for comparison with extracts of *Penicillium* species 27 was purchased from Merck. The ITS sequences are accessible at NCBI Genbank under the accession numbers ON303733–ON307363.

### Extraction and detection of NPs

Extraction and detection of NPs were carried out as described by Stroe and colleagues^22^. Briefly, the culture broth was homogenized using a rotor-stator homogenizer (ULTRA-TURRAX, IKA-Werke). The homogenized cultures were extracted twice with a total of 100 ml ethyl acetate, dried with sodium sulfate and concentrated under reduced pressure. For HPLC–MS analyses, the dried extracts were dissolved in 1 ml methanol and loaded onto an ultra-high-performance LC–MS system consisting of an UltiMate 3000 binary rapid-separation LC with a photodiode array detector (Thermo Fisher Scientific) and an LTQ XL linear ion trap mass spectrometer (Thermo Fisher Scientific) equipped with an electrospray ion source. The extracts (injection volume, 10 µl) were analysed on a 150 mm × 4.6 mm Accucore reversed-phase MS column with a particle size of 2.6 µm (Thermo Fisher Scientific) and the following solvent system: acetonitrile and distilled water (both supplemented with 0.1% (vol/vol) formic acid), a flow rate of 1 ml min^−1^ and gradient over 21 min (programme: initial 0% (vol/vol) acetonitrile increased to 80% (vol/vol) over 15 min and then to 100% (vol/vol) over 2 min, held at 100% (vol/vol) for 2 min and reversed to 0% (vol/vol) over 2 min). Identification of desertomycin A, monazomycin, lydicamycin and linearmycin was achieved by comparison with authentic references purchased from Santa Cruz Biotechnology and BioAustralis.

For the measurement of azalomycin F in both the biomass and in the supernatant of an *A. nidulans* culture, the biomass was separated from the medium using Miracloth.

HPLC-high-resolution electrospray ionization-MS and MS/MS measurements were performed using a Q Exactive Orbitrap high-performance benchtop LC–MS with an electronspray ion source and an UltiMate 3000 HPLC system with a photodiode array (Thermo Fisher Scientific) employing a C18 column (Accucore C18, 2.6 µm, 100 mm × 2.1 mm; Thermo Fisher Scientific) and the following solvent system: acetonitrile and distilled water (both supplemented with 0.1% (vol/vol) formic acid), a flow rate of 0.2 ml min^–1^, a gradient of 5–98% (vol/vol) acetonitrile for the first 10 min and hold until 14 min in 98% (vol/vol) acetonitrile. Compounds were identified by comparison with authentic references (retention time, ultraviolet light spectrum, and HR-MS and MS/MS spectra).

### MALDI-IMS

Co-cultures were set up by the addition of 3 ml AMM agar supplemented with 5 mM l-arginine, 1 ml l^−1^ trace elements, 0.3 mM FeSO_4_ and 3 µg ml^−1^ PABA directly on indium-tin oxide-coated glass slides. *A. nidulans* spores (*n* = 500) were spot inoculated and incubated at 37 °C overnight. An *S. iranensis* preculture was washed in PBS, 15 µl was spot inoculated 1 cm apart from the inoculation site of *A. nidulans* and the samples were incubated at 28 °C. After 2, 7 and 10 d two slides carrying an *S. iranensis* monoculture and an *A. nidulans–S. iranensis* co-culture were dried at 37 °C in a hybridization oven for 24 h. The dried samples were then sprayed with a saturated solution (20 mg ml^−1^) of universal MALDI matrix (1:1 mixture of 2,5-dihydroxybenzoic acid and α-cyano-4-hydroxy-cinnamic acid; Bruker Daltonics) prepared in acetonitrile/methanol/water (70:25:5, vol/vol/vol) using the automatic system ImagePrep device 2.0 (Bruker Daltonics) in 60 consecutive cycles (the sample was rotated 180° after 30 cycles) of 41 s (1 s spraying, 10 s incubation time and 30 s of active drying) similar to Hoffmann and Dorrestein^66^. The samples were analysed using an UltrafleXtreme MALDI TOF/TOF system (Bruker Daltonics), which was operated in positive reflector mode using flexControl 3.0. The analysis was performed in the 100–3,000 Da range with 30% laser intensity (laser type 4), accumulating 1,000 shots by taking 50 random shots at every raster position. Raster width was set at 200 µm. Calibration of the acquisition method was performed externally using Peptide calibration standard II (Bruker Daltonics) containing Bradykinin 1–7, Angiotensin II, Angiotensin I, Substance P, Bombesin, ACTH clip1–17, ACTH clip18–39 and Somatostatin 28. Spectra were processed with baseline subtraction in flexAnalysis 3.3 and aligned using several endogenous peaks (compounds present in the culture media). Processed spectra were uploaded in flexImaging 3.0 for visualization and SCILS Lab 2015b for analysis and representation. Chemical images were obtained using total ion count normalization and weak denoising.

### Production and purification of azalomycin F from *S. iranensis*

To obtain the azalomycin F complex from *S. iranensis*, 50 ml TSBY was inoculated with 5 × 10^8^ spores and the bacteria were incubated for 4 d at 28 °C with shaking at 180 r.p.m. The entire culture was centrifuged to separate the biomass from the culture supernatant. After lyophilization of the biomass, the content of the pellet was extracted twice with 50 ml methanol at 60 °C. Both extraction solutions were combined, dried through evaporation under reduced pressure and dissolved in 2 ml of 75% (vol/vol) aqueous methanol. The extract was subsequently loaded onto an *N*-vinylpyrrolidone-divinylbenzene copolymer resin SPE column (Macherey–Nagel), washed with 50% (vol/vol) aqueous methanol and eluted with 70% (vol/vol) aqueous methanol to obtain purified azalomycin F complex.

### Generation of *S. iranensis* deletion strains

Gene deletions in *S. iranensis* were generated using the lambda red system according to Netzker and colleagues^67^. Briefly, the genes to be deleted as well as the 2 kb upstream and downstream of the gene were PCR amplified using primers with added restriction sites. The genes and their flanking regions were cloned into pKOSi and the generated plasmids were then used to transform *Escherichia coli* DH10β. Next, the plasmids were transformed into *E. coli* BW25113 pIJ790 and the genes to be deleted were replaced by the gene *aac(3)IV*, conferring apramycin resistance and oriT. Recombination was carried out using a DNA template obtained by PCR of the *aac(3)IV* gene, oriT and homologous sites at their ends generated by specific primers. The resulting plasmids were transformed into non-methylating *E. coli* ET12567 pUZ8002 that was used for conjugation with *S. iranensis*. The conjugation and subsequent steps were carried out as described by Netzker and colleagues^67^. The deletion mutants were verified by Southern blot analysis (Supplementary Fig. 1a–c), essentially according to Southern^68^. Bacterial genomic DNA was isolated using a NucleoSpin microbial DNA mini kit (Macherey–Nagel). The oligonucleotide sequences used are listed in Supplementary Table 5. For Southern blot analysis, the enzymes used to cleave chromosomal DNA were BamHI (New England Biolabs) for verification of *ΔbldA*, BstEII (New England Biolabs) for Δ*bldD* and PstI (New England Biolabs) for *ΔbldH*.

### Generation of the *A. nidulans orsA*p-*nLuc*-*GFPs* reporter strain and screening for bacterial isolates inducing GFPs-dependent fluorescence

To study the activation of the *ors* BGC in *A. nidulans*, the human codon-optimized nanoluciferase (*nLuc*) gene (Promega) and the *GFPs* gene were translationally fused and placed under the control of the native *orsA* promoter by integrating the gene fusion and the *pabaA* gene as a selection marker in locus upstream of the *orsA* gene. The transformation cassette was constructed as previously described^69^. Approximately 2,000-bp sequences homologous to the regions upstream and downstream of *orsA* (*AN7909*) were amplified using the primer pairs MM025 and MM026, and MM030 and MM031, respectively. The *GFPs* and *nLuc* genes were amplified using the primer pair MM027 and MM037, thereby creating a 30-bp overhang homologue to the upstream region of the *orsA* gene. The *pabaA* gene was amplified using the primers MM061 and MM062, thereby creating a 3′-end sequence homologue to the *GFPs* gene and a 5′-end sequence homologue to the downstream region of the *orsA* gene. The pUC18 vector was amplified with MM033 and MM032, creating a 3′-end sequence homologous to the downstream and a 5′-end sequence homologous to the upstream region of the *orsA* gene. PCR products were assembled using the NEBuilder HiFi DNA assembly master mix (New England Biolabs) according to the manufacturer’s instructions and transformed into competent *E. coli* DH10β cells. The isolated and sequenced plasmid was PCR amplified with the primers MM025 and MM031 before transformation of *A. nidulans* RMS011. Transformation of *A. nidulans* was based on the protocol previously described by Balance and Turner^70^, with slight changes. Briefly, the biomass of a 16-h *A. nidulans* culture in AMM medium was harvested using Miracloth and added to osmolarity medium (1.2 M MgSO_4_, 3 mM Na_2_HPO_4_ and 7 mM NaH_2_PO_4_, pH 5.8) containing 10% (wt/vol) vinotaste (Novozymes). The protoplasts were harvested after 2 h at 30 °C with shaking at 80 r.p.m. by filtration through Miracloth and centrifugation at 1,016*g* for 10 min. The protoplasts were then washed twice with 10 mM Tris–HCl (pH 7.0) and 0.6 M KCl, and once with 0.6 M KCl, 50 mM CaCl_2_·2H_2_O and 10 mM Tris–HCl (pH 7.5), and resuspended in the latter. The subsequent protocol followed the description by Balance and Turner^70^ with the exception that 3 µg of PCR product was used instead of 1 µg plasmid DNA and AMM agar plates containing 5 mM l-arginine, 1 ml l^−1^ trace elements, 0.3 mM FeSO_4_ and 44.7 g l^−1^ KCl were used. Colonies of transformants were selected on AMM agar plates supplemented with 5 mM l-arginine, 1 ml l^−1^ trace elements and 0.3 mM FeSO_4_. The genomic structure of the transformant strain was verified by Southern blot analysis as described by Brakhage and van den Brulle^56^ using a probe directed against the *orsA* gene (Extended Data Fig. 6b), which was amplified using the primers MM031 and MM065 (Supplementary Table 5).

For the evaluation of the ability of bacteria isolated from soil to activate *orsA* that is indicated by green fluorescence and activity of the nanoluciferase, the *A. nidulans orsA*p-*nLuc*-*GFPs* strain was cultured in AMM medium supplemented with 5 mM l-arginine, 1 ml l^−1^ trace elements and 0.3 mM FeSO_4_. Mycelia of the overnight culture (approximately 16 h old) in AMM were separated from the medium using Miracloth and distributed in a 24-well plate (Greiner bio-one, Cellstar, Merck) and supplemented with 1 ml fresh AMM containing 5 mM l-arginine, 1 ml l^−1^ trace elements and 0.3 mM FeSO_4_. After 6 h of incubation at 37 °C with shaking at 600 r.p.m. in a tabletop shaker (Thermo-shaker PST-60HL, Biosan), fluorescence and bright-field images at ×4 magnification were taken using a Keyence BZ-X800 microscope.

For quantification of nLuc activity, the fungal mycelium was added to a 2 ml screwcap tube, filled with Zirconia beads (Thermo Fisher Scientific), and then homogenized by two rounds of 30 s (Speedmill Plus, Analytik Jena). After centrifugation, the luminescence of the supernatant was analysed using the Nano-Glo luciferase assay system (Promega) according to the manufacturer’s instructions using a microtiter plate reader (TECAN).

### RNA isolation, complementary DNA library construction and sequencing

Total RNA of *S. iranensis* WT and its deletion mutants was isolated using a Direct-zol RNA MiniPrep plus purification kit (Zymo Research Europe). Samples were taken after 48 h of cultivation in TSB medium. Bacterial cells were disrupted by bead beating for 4 min (Speedmill Plus, Analytik Jena). DNase treatment using Baseline-ZERO DNase (Lucigen) was followed by an RNA Clean & Concentrator-5 (Zymo Research Europe) clean-up procedure. For each sample, total RNA from three replicates was pooled and 2–3 µg RNA was processed for the library preparation. Library construction, Illumina NextSeq 500 paired-end sequencing, mapping and normalizing of the reads were performed by StarSEQ GmbH. Reads were aligned to the NCBI reference genome for *S. iranensis* (assembly GCA_000938975.1). Transcripts were normalized by counting the number of transcripts per million^71^. The RNA-sequencing data can be found at Gene Expression Omnibus under the accession number GSE201630.

### Protein extraction from *S. iranensis* and proteomics

Proteins were isolated from *S. iranensis* as follows. The collected biomass was dried, washed with sterile water and frozen in a mortar with liquid nitrogen. The frozen biomass was thoroughly ground using a mortar and pestle. The powdered biomass was added to 300 µl lysis buffer (10 ml lysis buffer consisted of 1% (wt/vol) SDS (100 mg), 150 mM NaCl, 100 mM triethyl ammonium bicarbonate (TEAB) and one tablet of cOmplete ultra protease inhibitor cocktail/phosSTOP (Roche)). Next, 4 µl benzonase (25 U µl^−1^; Merck Millipore) was added and the sample was incubated in a water-bath sonicator for 30 min at 37 °C. To collect the supernatant, the cells were centrifuged for 15 min at 18,000*g* and 4 °C, and the supernatant was transferred to another tube. The protein content was measured using a Merck Millipore direct detect system. The protein (100 µg) was transferred to an empty centrifuge tube, to which 100 mM TEAB was added to a final volume of 100 µl. A 2-µl volume of reduction buffer (500 mM tris(2-carboxyethyl)phosphine) was added to the sample and incubated at 55 °C for 1 h. After incubation, freshly prepared alkylation buffer (0.0584 g 2-chloroacetamide in 1 ml of 100 mM TEAB) was added. Next, 2 µl alkylation buffer (625 mM 2-chloroacetamide in 100 mM TEAB) was added and the sample was incubated at room temperature in the dark for 30 min. To precipitate the proteins, 400 µl chilled methanol (−20 °C) was added with thorough mixing, followed by the addition of 100 µl chilled chloroform (−20 °C) and mixing. Pure water (300 µl at 4 °C) was then added and the sample was thoroughly mixed to allow for phase separation. After an incubation at −20 °C of 5 min the samples were spun down (5 min at 14,000 r.p.m. (approximately 18,000*g*), <4 °C) and the aqueous layer was discarded, leaving the protein interphase intact. Next, the samples were washed in 400 µl chilled (−20 °C) methanol. The residual methanol was removed using a vacuum concentrator (Eppendorf). The proteins were dissolved in 100 µl of 100 mM TEAB. For digestion, 2 µl of a 1 µg µl^−1^ rLys C protein solution (Promega; 15 µg in 15 µl resuspension buffer (50 mM acetic acid); protease:protein, 1:50) was added, mixed and the sample was incubated for 2 h at 37 °C. Trypsin Gold protease (2 µl of a 2 µg µl^−1^ solution; 100 µg Trypsin Gold protease (Promega) in 50 µl resuspension buffer; protease:protein, 1:25) was added, mixed and incubated for 16 h at 37 °C. The reaction was stopped with 10 µl of 10% (vol/vol) formic acid. The peptides were dried in a vacuum concentrator (Eppendorf) and resolubilized in 25 µl of 0.05% (vol/vol) trifluoroacetic acid and 2% (vol/vol) acetonitrile in water. For HPLC–MS measurements, the peptide solution was filtered through a 0.2-µm spin filter at 14,000 r.p.m. (approximately 18,000*g*) for 15 min at 4 °C. The filtrate was transferred to HPLC vials. Mass spectrometry analysis was performed on a Q Exactive Plus instrument (Thermo Fisher Scientific) at a resolution of 140,000 and 17,500 full width at half maximum for MS1 and MS2 scans, respectively. Tandem mass spectra were searched against the NCBI database of *S. iranensis* (accessed 24 January 2019). A strict false discovery rate < 1% (peptide and protein level) and at least a search engine threshold >30 (Mascot), >4 (Sequest HT) or >300 (MS Amanda) were required for positive protein hits. Label-free protein quantification was based on the Minora algorithm of PD2.2 using a signal-to-noise ratio of >5. The MS proteomics data are accessible at the PRIDE archive under the project identifier PXD033242.

### Mapping of arginoketide-producing bacteria

To search for arginoketide producers, we followed two approaches. We screened: (1) published data for regions where producers of azalomycin F, desertomycin A, monazomycin, linearmycin A and lydicamycin were found; and (2) available genome sequences for the presence of genes encoding the arginine-loading domain required for azalomycin F biosynthesis (Fig. 3b). Numerous actinomycetes met one or both of the criteria. These actinomycetes had been sampled all over the world on virtually all continents, which underlines their ubiquitous distribution (Fig. 3b and Extended Data Table 2).

### Reverse transcription with quantitative PCR

Expression of the *orsA* gene was quantified using the primers orsA_FW and orsA_RV. After separating fungal biomass from the supernatant (Miracloth), the biomass was homogenized for 30 s (SpeedMill PLUS, Analytik Jena), followed by isolation of the total RNA (GeneMATRIX universal RNA purification kit, EURx; yeast RNA purification protocol, starting with step 4). DNase treatment was performed on-column with Baseline-ZERO DNase (Lucigen). RNA quantification and quality control were done using Qubit 4 RNA BR and IQ assay kits (Thermo Fisher Scientific), respectively. Reverse transcription of 2 µg RNA was performed with Maxima H^−^ reverse transcriptase (Thermo Fisher Scientific) for 3 h at 48 °C. For each sample, 10 ng cDNA was used for amplification (MyTaq HS Mix, Meridian Bioscience) to obtain EvaGreen (Biotium)-labelled PCR fragments. The cycling parameters included an initial DNA denaturation step at 95 °C for 2 min, followed by 40 cycles with DNA denaturation at 95 °C for 5 s and primer annealing and extension at 62 °C for 15 s. The size of the PCR amplicon for *orsA* was 92 bp. Controls with no added template were included to exclude primer dimers from interfering with amplification detection. The quantitative PCR results were analysed using the QuantStudio Design and Analysis software (version 1.5.2; Applied Biosystems). Relative gene expression was calculated using the ΔΔ*C*_t_ method, normalized to the expression of the *A. nidulans* γ-actin gene *AN6542* as the internal standard (primers actin FW and RV) using the formula $2^{-(C_t orsA - C_t AN6542)}$ and compared with an *A. nidulans* monoculture as the calibrator.

### Phylogeny and ANI

Phylogeny of the bacterial genomes was investigated using the Type Strain Genome Server provided by DSMZ (https://tygs.dsmz.de/). The ANI Calculator (https://www.ezbiocloud.net/tools/ani)^72^ was used to calculate ANI values.

### Statistics and reproducibility

All HPLC–MS chromatograms depicted display representative chromatograms from at least three independent replicates.

### Reporting summary

Further information on research design is available in the Nature Portfolio Reporting Summary linked to this article.

### Supplementary information

Supplementary Information Supplementary Figs. 1–8 (including captions and legends), Supplementary Tables 1–5, source data for Supplementary Fig. 1.

Reporting Summary

### Source data

Source Data Extended Data Fig. 2 Detailed qPCR results.

Source Data Extended Data Fig. 6 Unprocessed Southern blot.

## Figures and Tables

**Extended Data Table 1:**
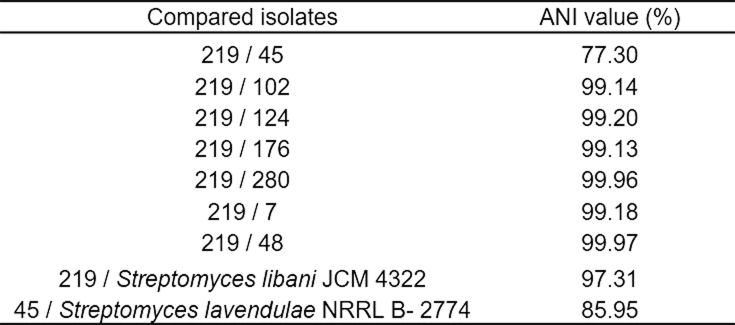
ANI values calculated by comparison of isolated *Streptomyces* strains 219 and 45 with other isolates and most closely related *Streptomyces* species

**Extended Data Table 2:**
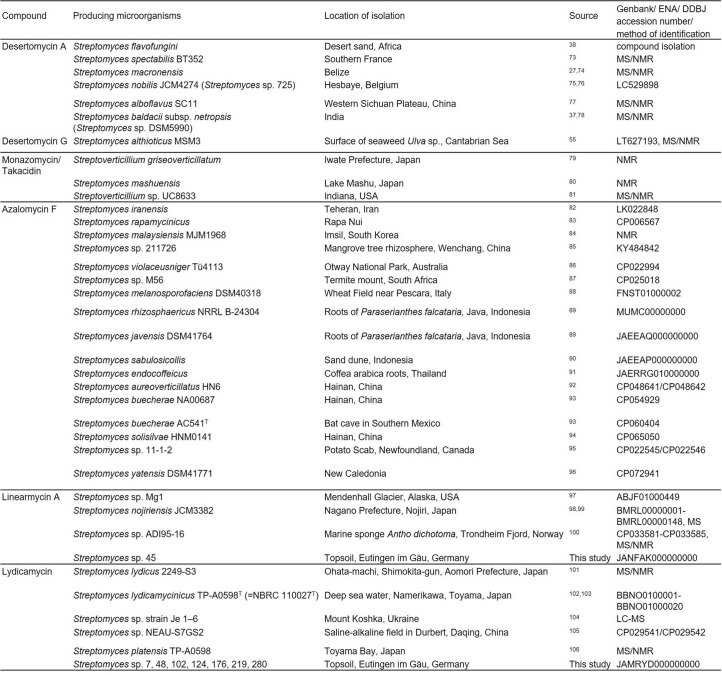
Worldwide occurrence of arginoketide-producing actinomycetes

## Data Availability

The authors declare that all data supporting the findings of this study are available within this article, its supplementary information, source data or in public repositories. The RNA-sequencing data can be accessed at the Gene Expression Omnibus (accession number GSE201630). DNA sequencing data can be found under NCBI BioProject PRJNA830323. The MS proteomics data are available at PRIDE under the dataset identifier PXD033242. The ITS sequences are available under the NCBI accession numbers ON307333–ON307363. Source data are provided with this paper.
