# Supplementary material for: Streptomyces polyketides mediate bacteria–fungi interactions across soil environments
Source: Nat Microbiol. 2023 Jun 15;8(7):1348–61. doi: 10.1038/s41564-023-01382-2 (PMC10322714; doi:10.1038/s41564-023-01382-2)
Supplement: Supplementary file 1 — Supplementary Figs. 1–8 (including captions and legends), Supplementary Tables 1–5, source data for Supplementary Fig. 1. [file 41564_2023_1382_MOESM1_ESM.pdf]

nature microbiology

Article https://doi.org/10.1038/s41564-023-01382-2

Streptomyces polyketides mediate bacteria–fungi interactions across soil environments

In the format provided by the authors and unedited

Table of contents:

Supplementary Figures

Supplementary Figure 1: Southern blot analysis of the generated *S. iranensis* deletion mutants.

Supplementary Figure 2: Azalomycin F specifically associates with fungal biomass.

Supplementary Figure 3: Wild-type *A. nidulans* FGSC A4 produces orsellinic acid, lecanoric acid, and F-9775A/B when treated with azalomycin F.

Supplementary Figure 4: Rapamycin is not involved in the induction of the production of fungal orsellinic acid derivatives.

Supplementary Figure 5: Comparison of the published BGCs for lydicamycin and linearmycin with the genomic information for the soil isolates *Streptomyces* sp. 219 and 45.

Supplementary Figure 6: HR-MS and MS/MS analyses. Culture extracts were compared to commercial references.

Supplementary Figure 7: Identification of fungal soil isolates differently responding to *S. iranensis* WT and the azalomycin F-deficient Δ*azlH* mutant strain.

Supplementary Figure 8: HPLC-MS analysis of four monocultures and cocultures of *Penicillium* sp. soil isolates with *S. iranensis* WT and *S. iranensis* Δ*azlH*.

Supplementary Tables

Supplementary Table 1: AntiSMASH-annotated BGCs downregulated in *S. iranensis* Δ*bldA* compared to the WT.

Supplementary Table 2: Comparison of the lydicamycin BGC of *Streptomyces* sp. ID38640 with sequences of bacterial soil isolate 219.

Supplementary Table 3: Comparison of the linearmycin BGC of *Streptomyces sp*. Mg1 with sequences of bacterial soil isolate 45.

Supplementary Table 4: Microorganisms and plasmids, their genotype and reference.

Supplementary Table 5: Primers used in this study.

Source Data

Source Data for Supplementary Figure 1: Uncropped Southern Blots verifying successful deletion of the *bld* genes.

Supplementary Figures

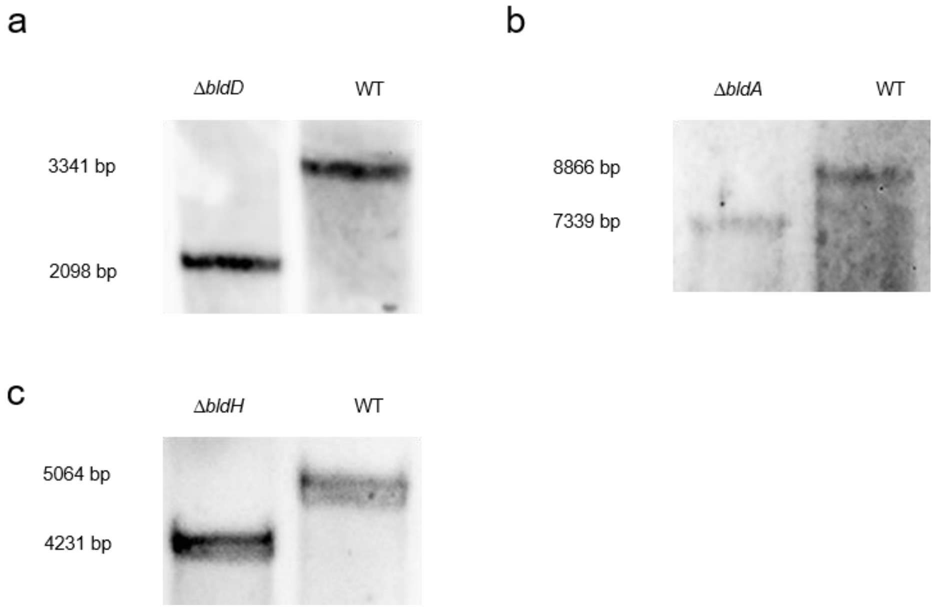

Supplementary Fig. 1: Southern blot analysis of the generated *S. iranensis* deletion mutants. Each blot shows the band characteristic of the indicated deletion mutant in comparison to a band obtained with *S. iranensis* WT. Genomic DNA was digested with *Bam*HI for verification of *ΔbldA*, *Bst*EII for Δ*bldD*, and *Pst*I for *ΔbldH*. Each blot was performed once.

a, Deletion of *bldD*. The expected band for a deletion has a size of 2098 bp, for the WT 3341 bp. **b,** Deletion of *bldA.* Deletion of *bldA* is indicated by a band of 7339 bp, for the WT 8866 bp. **c,** Deletion of *bldH.* Insertion of the resistance cassette shows a band of 4231 bp, for the wild type 5064 bp.

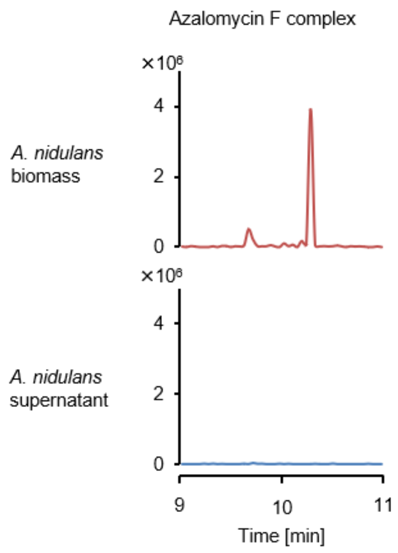

Supplementary Fig. 2: Azalomycin F specifically associates with fungal biomass. Extracted ion chromatogram of azalomycin F3a (*m/z* 1067 $[M\text{-}H]^-$) derived from LC-MS analyses of biomass and culture supernatant of an *A. nidulans* culture supplemented with azalomycin F.

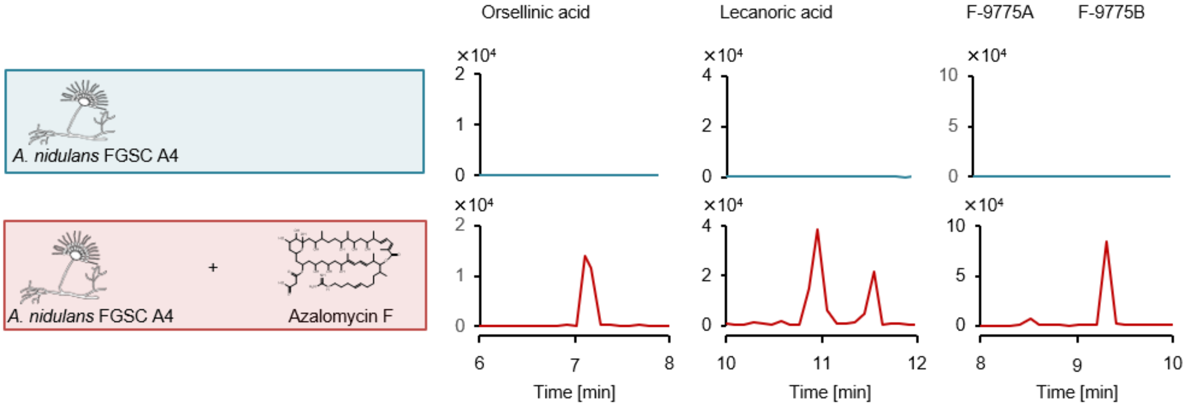

Supplementary Fig. 3: Wild-type *A. nidulans* FGSC A4 produces orsellinic acid, lecanoric acid, and F-9775A/B when treated with azalomycin F. Fungal cultures were treated with 10 µg/mL azalomycin F. Control cultures were not treated with the compound. Extracted ion chromatograms for orsellinic acid (*m/z* 167 $[M-H]^-$), lecanoric acid (*m/z* 317 $[M-H]^-$) and F-9775A and F-9775B (*m/z* 395 $[M-H]^-$) derived from LC-MS analysis of culture extracts.

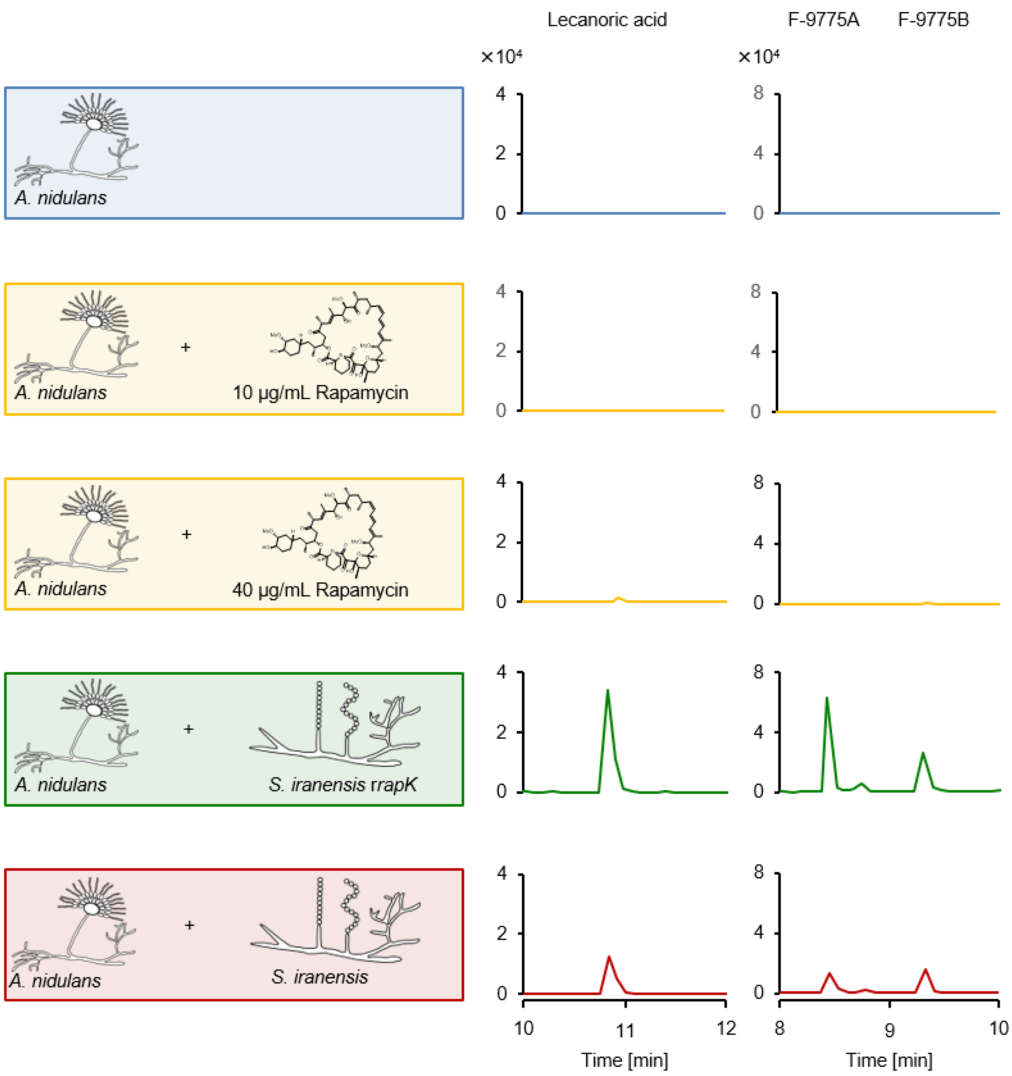

Supplementary Fig. 4: Rapamycin is not involved in the induction of the production of fungal orsellinic acid derivatives.

Cultivation of *A. nidulans* with rapamycin or cocultivation of *A. nidulans* with the rapamycin biosynthesis mutant Δ*rapK* of *S. iranensis* and the WT. Extracted ion chromatograms for orsellinic acid dimer lecanoric acid (*m/z* 317 $[M-H]^-$) and F-9775A and F-9775B (*m/z* 395 $[M-H]^-$) derived from LC-MS analysis of culture extracts.

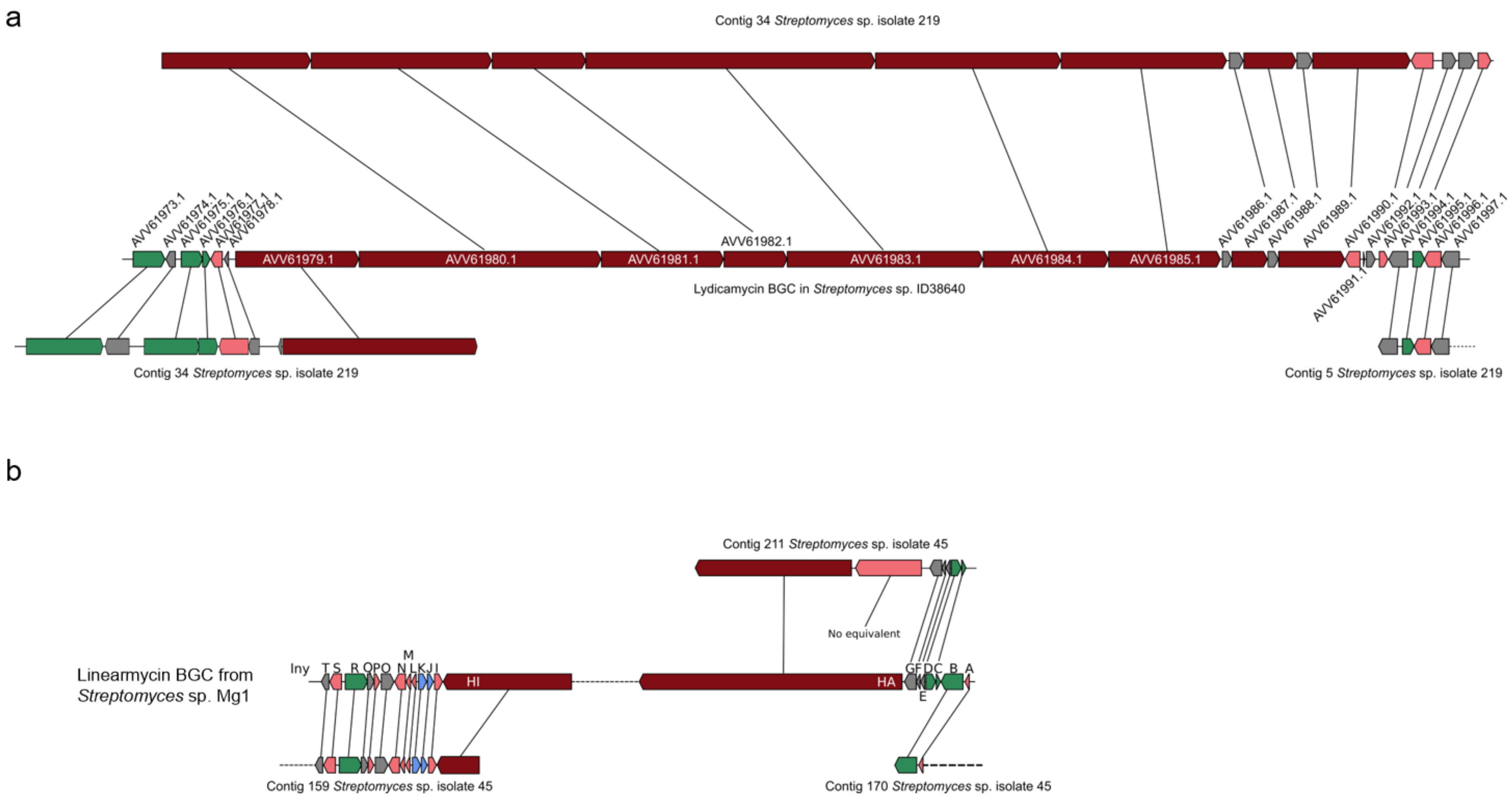
a
Contig 34 Streptomyces sp. isolate 219
AVV61973.1
AVV61974.1
AVV61975.1
AVV61976.1
AVV61977.1
AVV61978.1
AVV61979.1
AVV61980.1
AVV61981.1
AVV61982.1
AVV61983.1
AVV61984.1
AVV61985.1
AVV61986.1
AVV61987.1
AVV61988.1
AVV61989.1
AVV61990.1
AVV61991.1
AVV61992.1
AVV61993.1
AVV61994.1
AVV61995.1
AVV61996.1
AVV61997.1
Lydicamycin BGC in Streptomyces sp. ID38640
Contig 34 Streptomyces sp. isolate 219
Contig 5 Streptomyces sp. isolate 219
b
Contig 211 Streptomyces sp. isolate 45
No equivalent
Linearmycin BGC from Streptomyces sp. Mg1
lny
T S R Q P O N M L K J I
HI
HA
G F D C B A
E
Contig 159 Streptomyces sp. isolate 45
Contig 170 Streptomyces sp. isolate 45

Supplementary Fig. 5: Comparison of the published BGCs for lydicamycin and linearmycin with the genomic information for the soil isolates *Streptomyces* sp. 219 and 45.

a, The published BGC of lydicamycin from *Streptomyces* sp. ID38640 was compared to the illumina contigs of the genome of *Streptomyces* sp. isolate 219. The lydicamycin BGC in this isolate is found on three contigs, that in combination contain all genes of the lydicamycin BGC. Percentage of nucleotide-identity is shown in Supplementary Table 2.

b, Comparison of the published linearmycin BGC of *Streptomyces* sp. Mg1 with the genomic information of *Streptomyces* sp. isolate 45. Conserved genes for all genes encoding the tailoring enzymes of the biosynthesis of linearmycin were found in *Streptomyces* sp. isolate 45 (*lnyA-G* and *lnyI-T*). Homologs of the polyketide synthase genes *lnyHA* and *lnyHI* were also identified. The core polyketide synthase genes could not be assigned to specific contigs since their repetitive sequences were apparently not resolved by illumina sequencing. Furthermore, *Streptomyces* sp. isolate 45 harbors an additional gene in its putative linearmycin BGC that is not found in *Streptomyces* sp. Mg1. Percentage of nucleotide-identity is shown in Supplementary Table 3.

a: **30-Demethyllydicamycin**

HRESI-MS: *m/z* 841.5322 $[M+H]^+$, calcd. $C_{46}H_{73}N_4O_{10}$ 841.5327

MS/MS spectrum of *m/z* 841, top: extract; bottom: reference

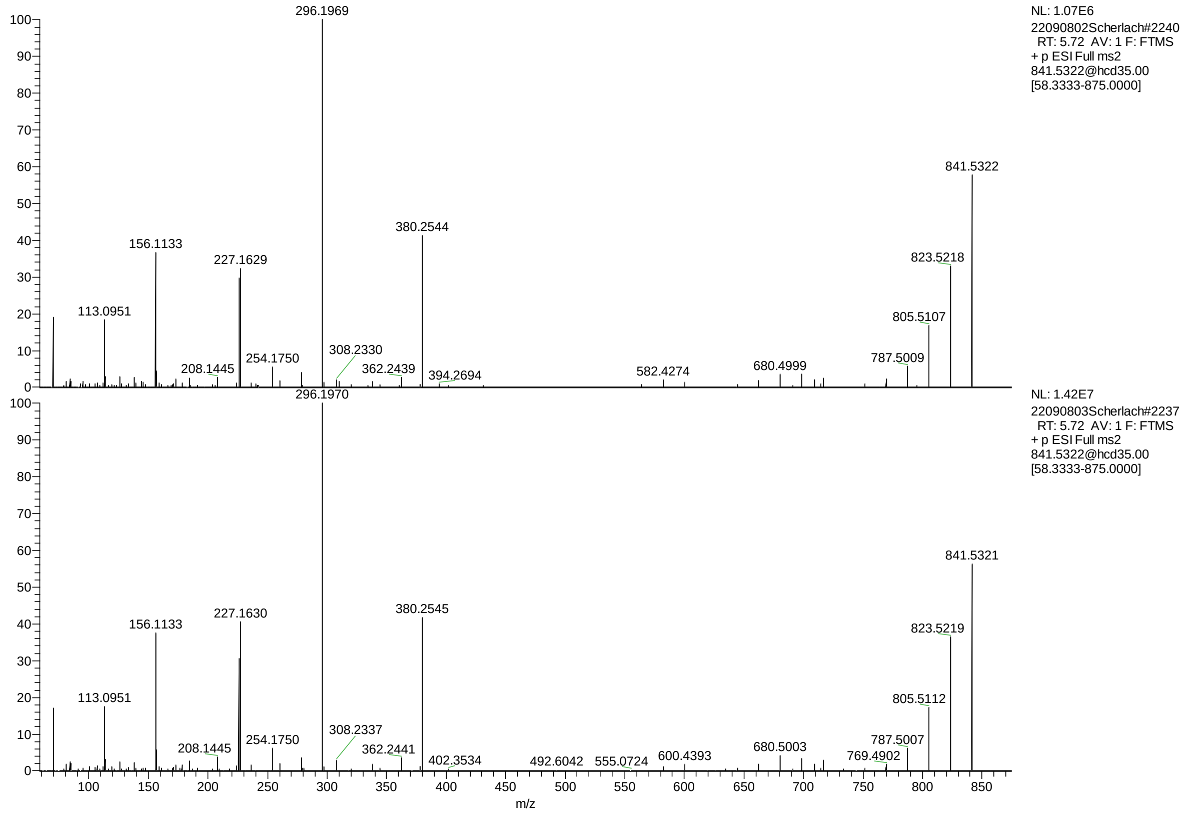

b: **Linearmycin derivative**

HRESI-MS: *m/z* 1140.7197 $[M+H]^+$, calcd. $C_{64}H_{102}NO_{16}$ 1140.7193

MS/MS spectrum of *m/z* 1140, top: extract; bottom: linearmycin A

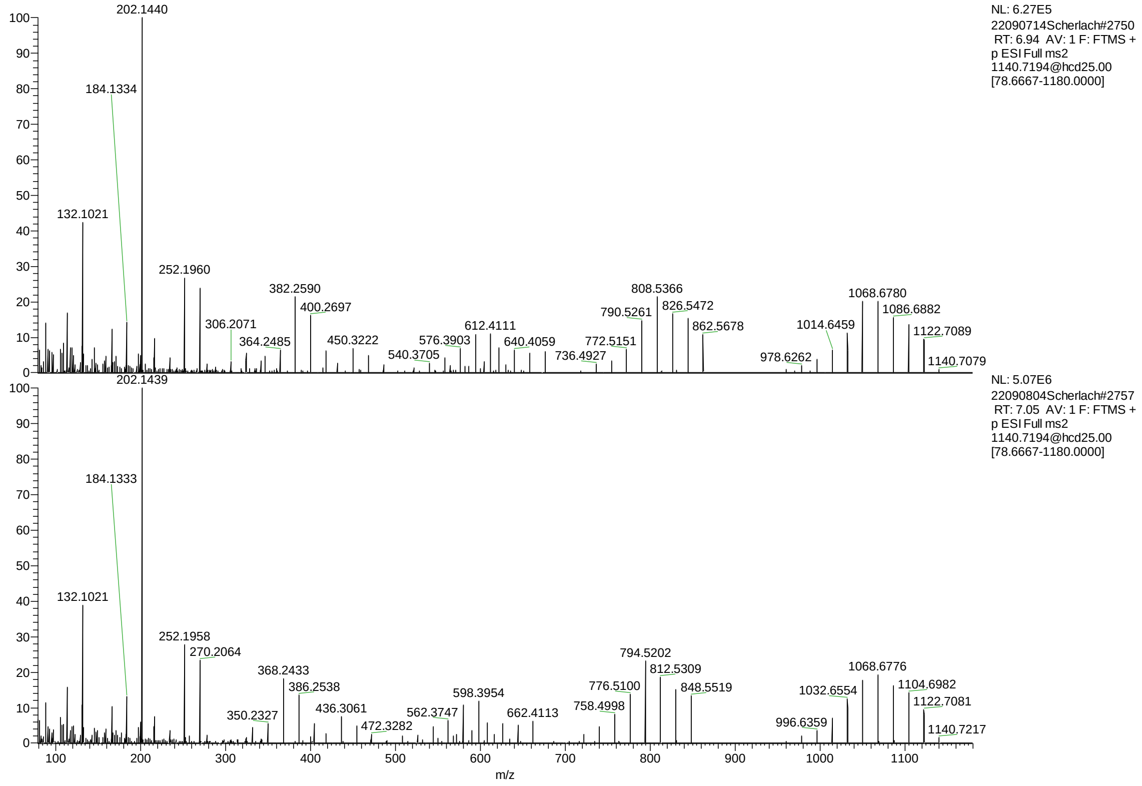

c: **Carviolin**

HRESI-MS: *m/z* 299.0557 [M-H]$^-$, calcd. $C_{16}H_{11}O_6$ 299.0550; *m/z* 301.0707 [M+H]$^+$, calcd. $C_{16}H_{13}O_6$ 301.0707

MS/MS spectrum of *m/z* 301, top: extract; bottom: reference

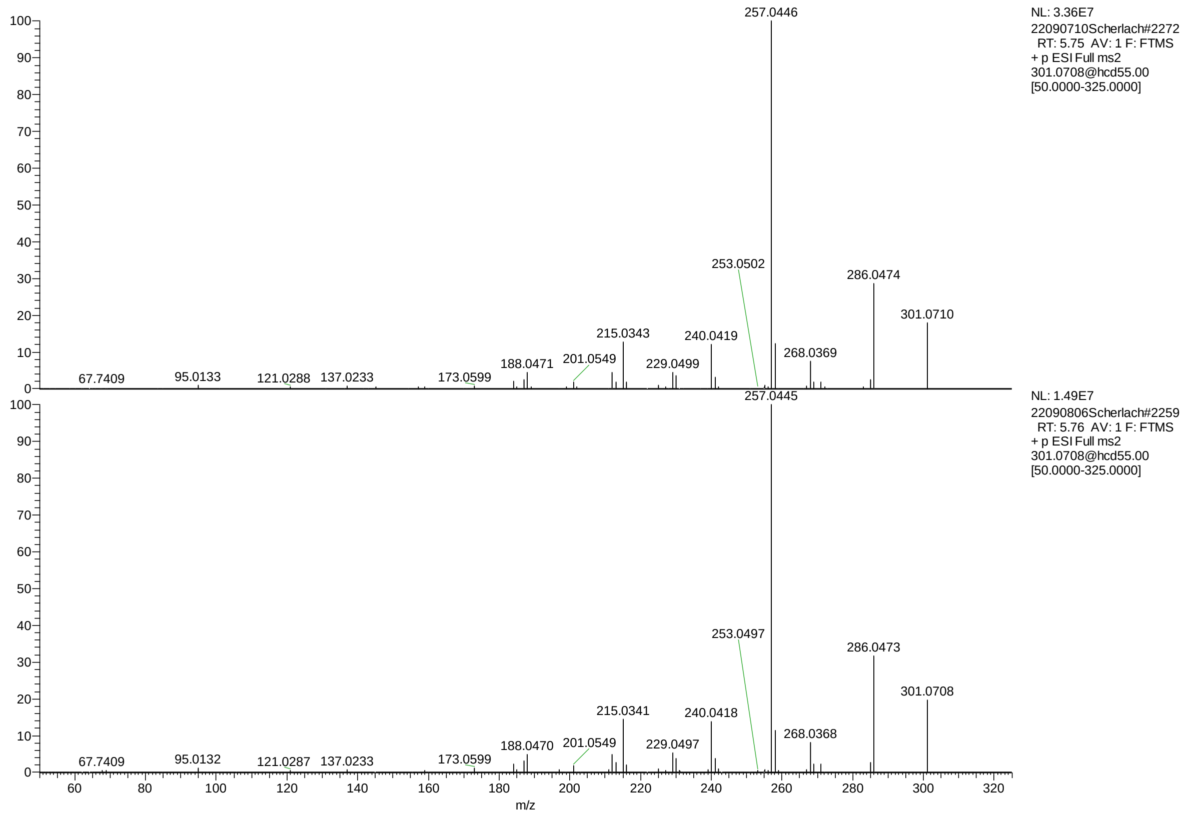

Supplementary Fig. 6: HR-MS and MS/MS analyses. Culture extracts were compared to commercial references.

a, HR-MS mass calculation and MS/MS analysis of 30-demethyllydicamycin reference and the culture extract of *Streptomyces* sp. isolate 219.

b, HR-MS mass calculation and MS/MS analysis of linearmycin A reference and the culture extract of *Streptomyces* sp. isolate 45.

c, HR-MS mass calculation and MS/MS analysis of carviolin reference and the culture extract of *Penicillium* sp. isolate 27 in coculture with *S. iranensis*.

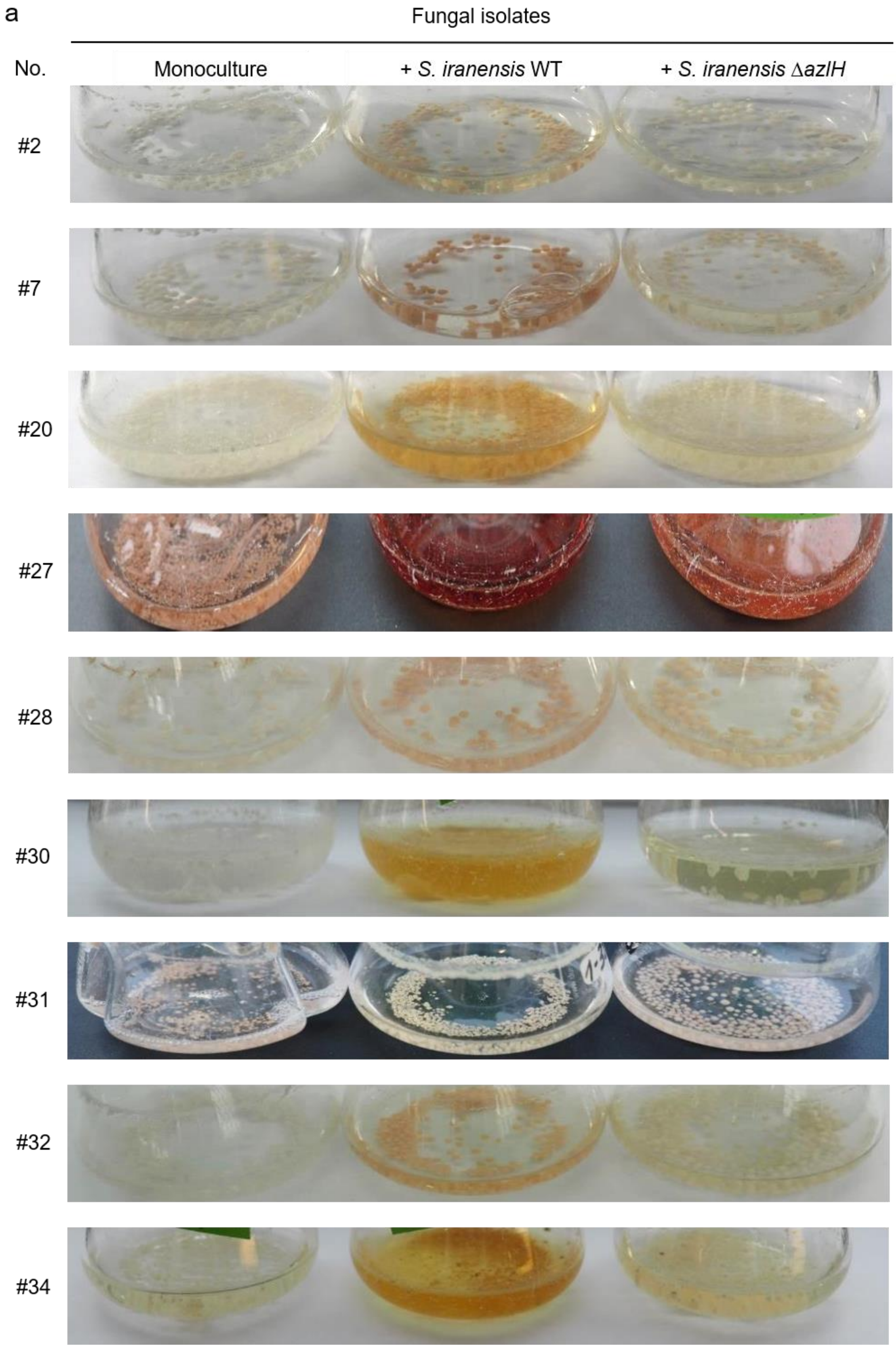
a
Fungal isolates
No.
Monoculture
+ S. iranensis WT
+ S. iranensis ΔazlH
#2
#7
#20
#27
#28
#30
#31
#32
#34

Fungal isolates

No.	Monoculture	+ *S. iranensis* WT	+ *S. iranensis* Δ*azlH*
#36			
#37			
#39			
#40			
#45			
#46			
#51			
#53			

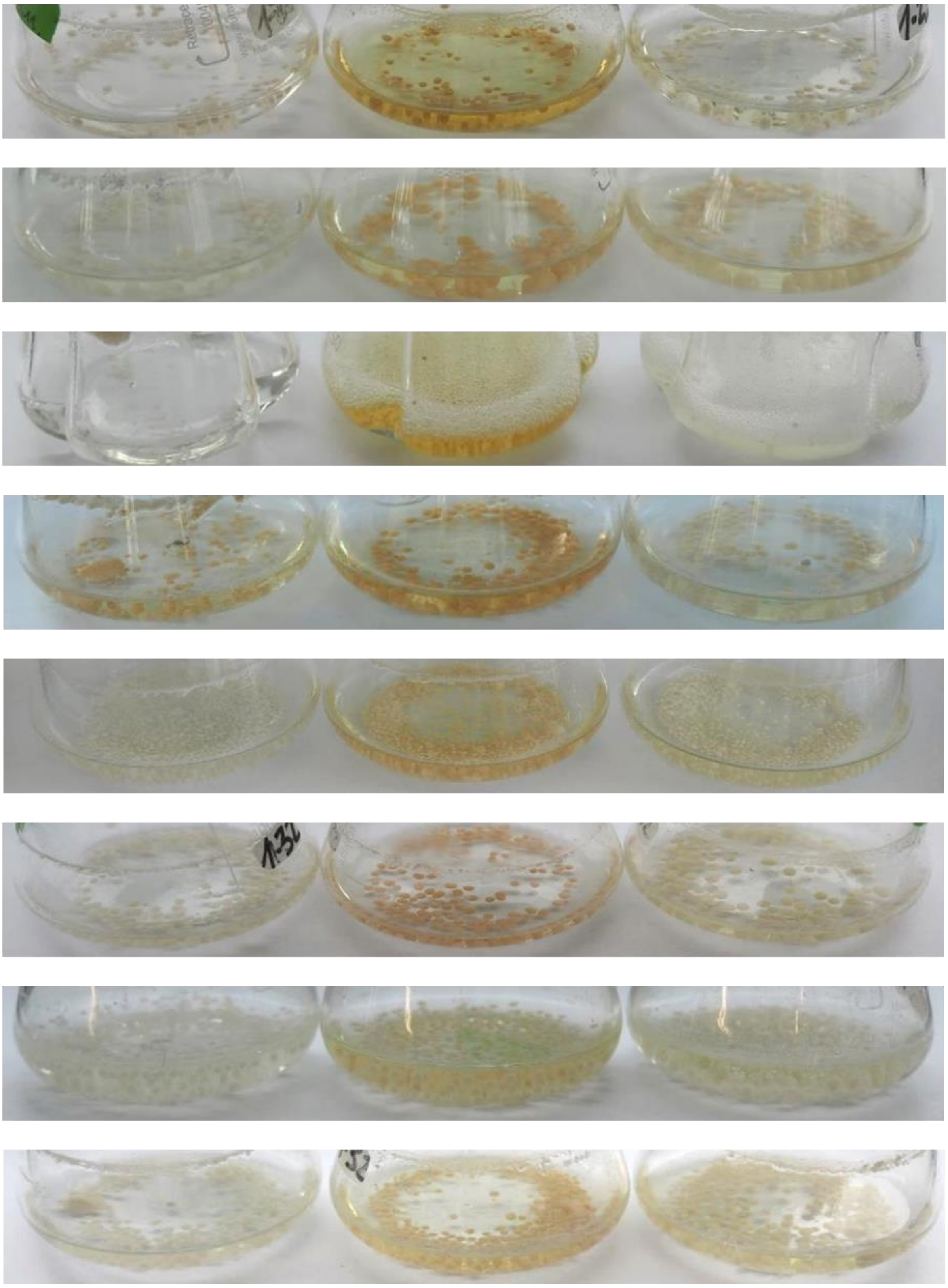

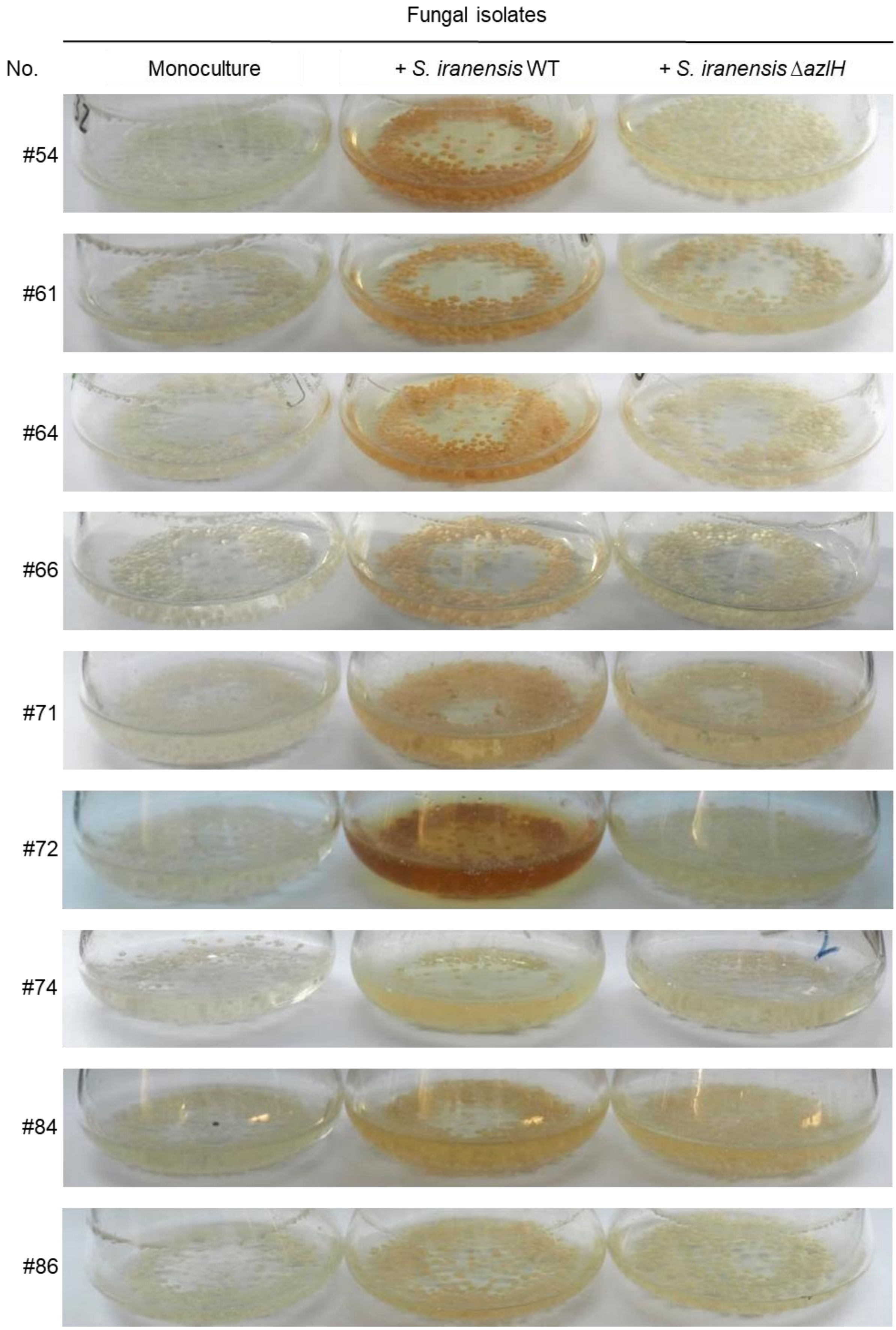
Fungal isolates
No.
Monoculture
+ *S. iranensis* WT
+ *S. iranensis* Δ*azlH*
#54
#61
#64
#66
#71
#72
#74
#84
#86

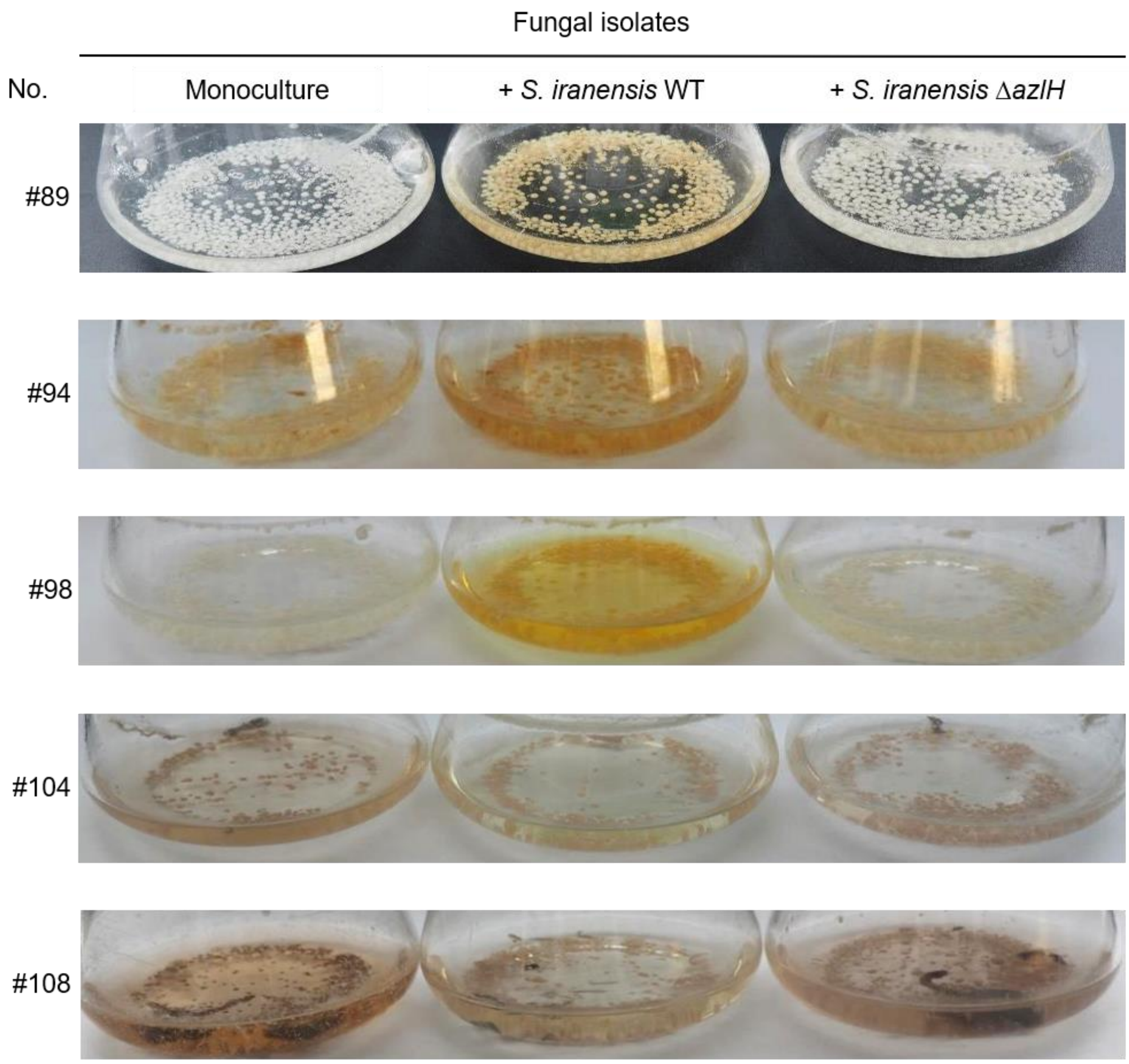
Fungal isolates
No.
Monoculture
+ S. iranensis WT
+ S. iranensis ΔazlH
#89
#94
#98
#104
#108

b

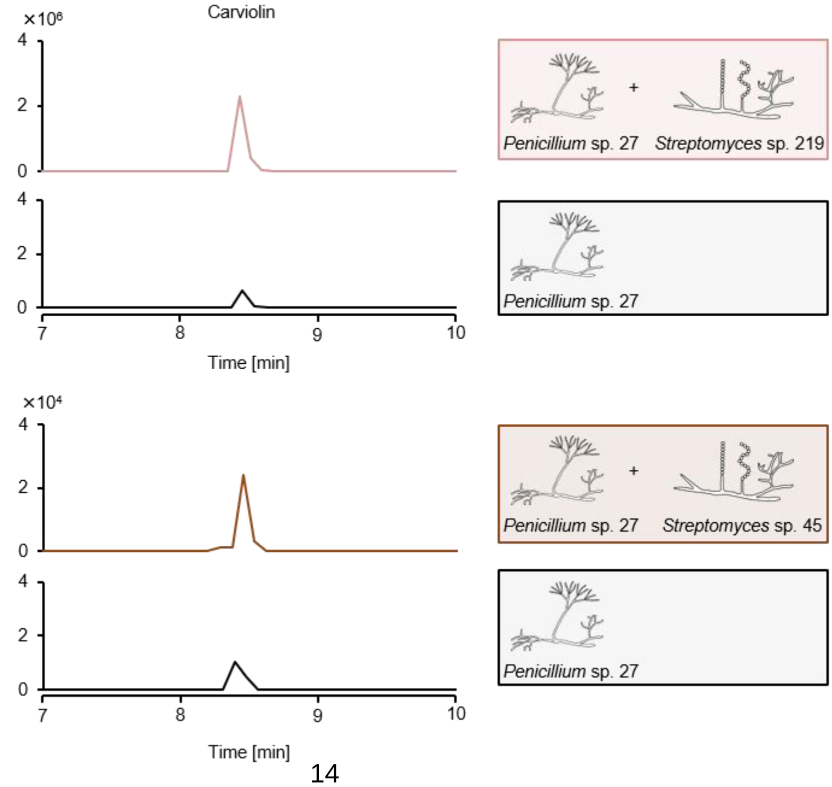
Carviolin
×10⁶
4
2
0
4
2
0
7
8
9
10
Time [min]
×10⁴
4
2
0
4
2
0
7
8
9
10
Time [min]
Penicillium sp. 27
+
Streptomyces sp. 219
Penicillium sp. 27
Penicillium sp. 27
+
Streptomyces sp. 45
Penicillium sp. 27

Supplementary Fig. 7: Identification of fungal soil isolates differently responding to *S. iranensis* WT and the azalomycin F-deficient ∆*azlH* mutant strain. a, Cultivation flasks with monocultures and cocultures of fungal soil isolates with *S. iranensis* WT and the azalomycin F-deficient mutant strain ∆*azlH* are shown. **b,** Extracted ion chromatograms for carviolin (*m/z* 299 $[M-H]^-$) derived from LC-MS analysis of culture extracts of mono- and cocultivation of *Penicillium* sp. 27 with *Streptomyces* sp. 45 and *Streptomyces* sp. 219.

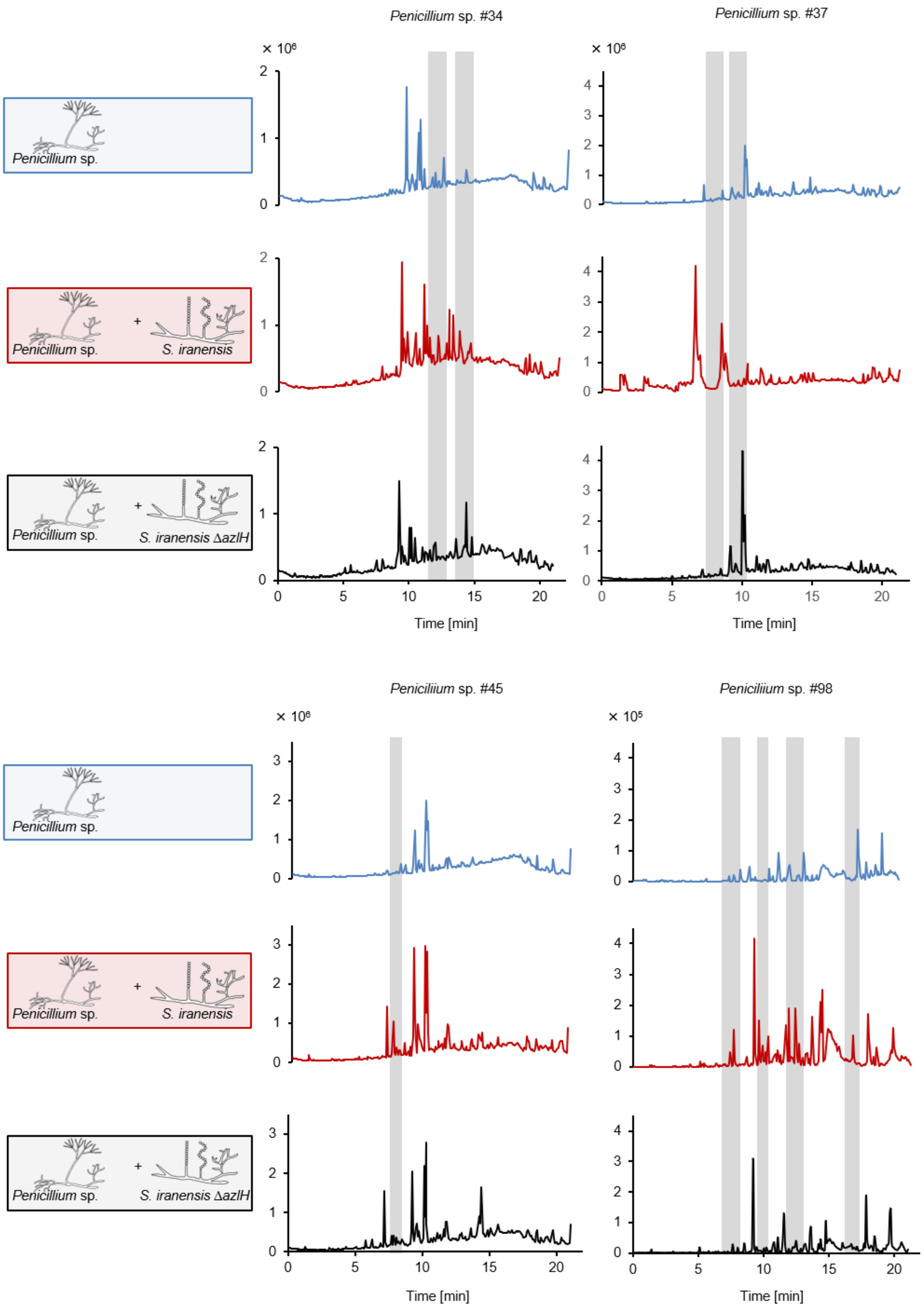

Supplementary Fig. 8: HPLC-MS analysis of four monocultures and cocultures of *Penicillium* sp. soil isolates with *S. iranensis* WT and *S. iranensis ΔazlH*.

Depicted are total ion chromatograms of monocultures of *Penicillium* sp. #34, #37, #45, and #98 as well as of their cocultures with *S. iranensis* WT and azalomycin F-deficient *S. iranensis* Δ*azlH* mutant strain. Grey bars highlight new mass peaks only observed for the coculture with the *S. iranensis* wild type, suggesting the exclusive production of NPs only in presence of this bacterium.

Supplementary Tables

Supplementary Table 1: AntiSMASH-annotated BGCs downregulated in *S. iranensis* Δ*bldA* compared to the WT.

Product and/or most similar BGC (%)	Gene ID	Annotation	mRNA steady state level log2 fold-change Δ*bldA* *vs.* WT
Funisamine (20 %)	*SIRAN_RS00110*	type I polyketide synthase	-4.08
	SIRAN_RS52200	acyltransferase domain-containing protein	-5.77
	SIRAN_RS52190	beta-ketoacyl synthase	
	SIRAN_RS00095	cytochrome P450	-6.72
	SIRAN_RS00075	acyltransferase domain-containing protein	-6.06
	SIRAN_RS00065	SDR family NAD(P)-dependent oxidoreductase	-5.59
	SIRAN_RS49670	type I polyketide synthase	-6.25
	SIRAN_RS00055	type I polyketide synthase	-5.47
	SIRAN_RS48495	type I polyketide synthase	-5.00
	SIRAN_RS52180	type I polyketide synthase	-5.01
	SIRAN_RS00045	type I polyketide synthase	-5.69
Butyrolactone/ A-factor	*SIRAN_RS03170*	hypothetical protein	-5.20
	SIRAN_RS03165	SDR family oxidoreductase	-5.80
	SIRAN_RS03155	A-factor biosynthesis protein	-5.54
Rapamycin	*SIRAN_RS38510*	type I polyketide synthase	-6.09
	SIRAN_RS38505	polyketide synthase	-6.35
	SIRAN_RS38490	type I polyketide synthase	-5.89
	SIRAN_RS38480	non-ribosomal peptide synthetase	-6.12
	SIRAN_RS53235	acyl carrier protein	-6.16
	SIRAN_RS38465	methyltransferase domain-containing protein	-6.58
	SIRAN_RS38460	ferredoxin	-3.09
	SIRAN_RS38455	cytochrome P450	-3.35
	SIRAN_RS38450	class I SAM-dependent methyltransferase	-4.67
	SIRAN_RS38440	dioxygenase	-6.27
	SIRAN_RS38435	C9 hydroxylase cytochrome P450	-6.27
	SIRAN_RS38385	non-ribosomal peptide synthetase	-5.40
	SIRAN_RS38380	thioesterase	-5.95

Caniferolides (12 %)	*SIRAN_RS44280*	TetR/AcrR family transcriptional regulator	0.49
	SIRAN_RS44275	SDR family oxidoreductase	-0.84
	SIRAN_RS44265	copper amine oxidase	-6.19
	SIRAN_RS44260	serine hydrolase	-7.91
	SIRAN_RS44250	non-ribosomal peptide synthetase	-4.22
	SIRAN_RS44245	MFS transporter	-4.23
	SIRAN_RS44225	cytochrome P450	-4.42
	SIRAN_RS44195	cytochrome P450	-4.61
	SIRAN_RS44180	SDR family oxidoreductase	-5.68
	SIRAN_RS44170	type I polyketide synthase	-4.26
	SIRAN_RS51860	type I polyketide synthase	-6.03
	SIRAN_RS44140	type I polyketide synthase	-5.38
	SIRAN_RS44135	cytochrome P450	-6.40
	SIRAN_RS44125	glucan biosynthesis protein	-3.91

Supplementary Table 2: Comparison of the lydicamycin BGC of *Streptomyces* sp. ID38640 with sequences of bacterial soil isolate 219. Homologous genes were found in bacterial soil isolate 219 for each gene of the lydicamycin BGC of *Streptomyces* sp. ID38640[1].

Genes of the lydicamycin BGC of *Streptomyces* sp. ID38640[1]	Percentage of identical nucleotides of bacterial soil isolate 219 to *Streptomyces* sp. ID38640[1].	Location
G7Z12_32710 (4-guanidinobutyryl-CoA biosynthesis)	97%	Contig 34
AVV61973.1	97%	Contig 34
AVV61974.1	97%	Contig 34
AVV61975.1	97%	Contig 34
AVV61976.1	98%	Contig 34
AVV61977.1 (4-guanidinobutyryl-CoA loading)	97%	Contig 34
AVV61978.1	98%	Contig 34
AVV61979.1	96%	Contig 34
AVV61980.1	94%	Contig 28<br>Contig 240<br>Contig 241<br>Contig 242
AVV61981.1	95%	Contig 28
AVV61982.1	96%	Contig 28
AVV61983.1	95%	Contig 28
AVV61984.1	97%	Contig 28
AVV61985.1	97%	Contig 28
AVV61986.1	98%	Contig 28
AVV61987.1	98%	Contig 28

AVV61988.1	98%	Contig 28
AVV61989.1	97%	Contig 28
AVV61990.1	97%	Contig 28
AVV61992.1	98%	Contig 28
AVV61993.1	97%	Contig 28
AVV61994.1	97%	Contig 5
AVV61995.1	97%	Contig 5
AVV61996.1 (4-guanidinobutyric acid biosynthesis)	97%	Contig 5
AVV61997.1	98%	Contig 5

Supplementary Table 3: Comparison of the linearmycin BGC of *Streptomyces sp*. Mg1[2] with sequences of bacterial soil isolate 45. All genes encoding the tailoring enzymes of the biosynthesis of linearmycin were found to be highly conserved in bacterial soil isolate 45 (*lnyA-G* and *lnyI-T*). Homologs of the polyketide synthase genes *lnyHA* and *lnyHI* were also identified. The core polyketide synthase genes could not be assigned to specific contigs since their repetitive sequences were apparently not resolved by illumina sequencing.

Linearmycin BGC	Percentage of identical nucleotides of bacterial soil isolate 45 to *Streptomyces* sp. Mg1[2].	Location
LnyA	85%	Contig 170
LnyB	86%	Contig 170
LnyC	91%	Contig 211
LnyD	85%	Contig 211
LnyE	86%	Contig 211
LnyF	90%	Contig 211
LnyG	90%	Contig 211
M444_03000 (outside *lyn* BGC)	88%	Contig 211
LnyHA	92%	Contig 211 (end of Contig)
LnyHB		
LnyHC		
LnyHD		
LnyHE		
LnyHF		
LnyHG		
LnyHH		
LnyHI (4-guanidinobutyryl-CoA loading)	85%	Contig 159 (end of Contig)
LnyI	92%	Contig 159

LnyJ	92%	Contig 159
LnyK	86%	Contig 159
LnyL	80%	Contig 159
LnyM	84%	Contig 159
LnyN (4-guanidinobutyryl-CoA biosynthesis)	89%	Contig 159
LnyO	88%	Contig 159
LnyP	86%	Contig 159
LnyQ	87%	Contig 159
LnyR	79%	Contig 159
LnyS	89%	Contig 159
LnyT	82%	Contig 159

Supplementary Table 4: Microorganisms and plasmids, their genotype and reference.

Organism/ plasmid	Relevant genotype and phenotype	Reference
**Microorganisms**		
Streptomyces iranensis DSM41954 (HM35^T)	Wild-type strain	[3]
Streptomyces iranensis Δ*azl4*Δ*azl5*	*SIRAN1022&SIRAN1023*:: *aac(3)IV*, *oriT,* Apra^R	[4]
Streptomyces iranensis Δ*azlH*	*azlH (SIRAN1025)*::*aac(3)IV*, *oriT,* Apra^R	[4]
Streptomyces iranensis Δ*bldA*	*SIRAN_RS27980*::*aac(3)IV*, *oriT,* Apra^R	This study
Streptomyces iranensis Δ*bldD*	*SIRAN2101*::*aac(3)IV*, *oriT,* Apra^R	This study
Streptomyces iranensis Δ*bldH*	*SIRAN6239*::*aac(3)IV*, *oriT,* Apra^R	This study
Streptomyces macronensis UC 8271 (NRRL12566)	Wild-type strain	[5]
Streptomyces mashuensis DSM40896	Wild-type strain	German Collection of Microorganisms and Cell Cultures (DSMZ),

		Braunschweig, Germany
Aspergillus nidulans RMS011	*pabaA1, yA2*; Δ*argB*::*trpC*Δ*B*; *veA1, trpC801*	[6]
*Aspergillus nidulans orsA*p-*nLuc-GFPs*	RMS011 background, *pabaA::orsAp- nLuc-GFPs*	This study
Aspergillus nidulans FGSC A4	Wild-type strain	Fungal Genetics Stock Center, Kansas, USA
Aspergillus fumigatus ATCC 46645	Wild-type strain, *MAT-1*	[7]
Aspergillus fumigatus CEA10	Wild-type strain	[8]
Escherichia coli DH10β	*Δ(ara-leu) 7697, araD139, fhuA, ΔlacX74, galK16, galE15, e14-, ϕ80dlacZΔM15, recA1, relA1, endA1, nupG, rpsL (StrR), rph spoT1, Δ(mrr-hsdRMS-mcrBC)*	New England Biolabs, Ipswich, MA, USA
Escherichia coli BW25113	*lacI*^{q}, *rrnB*$_{T14}$, Δ*lacZ*$_{WJ16}$, *hsdR*514, Δ*araBA*-, *D*$_{AH33}$, Δ*rhaBAD*$_{LD78}$	[9]
Escherichia coli ET12567	F^{-}, *dam*-13::Tn9, *dcm*-6, *hsdR*, *zij*-202 ::Tn10, *recF*143, *galK*2, *galT*22, *ara*-14, *lac*Y1, *xyl*-5, *leuB*6, *thi*-1, *tonA*31, *rpsL*136, *HisG*4, *tsx*-78, *mtl*-1, *glnV*44	[10]
**Plasmids**		
pMM12-*orsA*p-*nLuc-GFPs*	pUC18, *pabaA*, *orsA*p-*nLuc-GFPs*	This study
pIJ790	λ-RED (*gam*, *bet*, *exo*), *cat*, *araC*, rep101ts, CatR	[11]
pUZ8002	RK2 derivative with defective *oriT* (aph)	[12]
pKOSi	*kan*, pSG5 replicon, *oriT,* Kan^{R}	[13]
pKOSi_*bldD*	*kan*, RSF *ori*, pSG5, *bldD (SIRAN2101),* Kan^{R}	This study
pKOSi_Δ*bldD*	*kan*, RSF *ori*, pSG5, *bldD* (*SIRAN2101*)::*aac(3)IV*, *oriT,* Kan^{R}	This study

pKOSi_ *bldA*	*kan*, RSF *ori*, pSG5, *bldA* (*SIRAN_RS27980*), Kan^R	This study
pKOSi_Δ*bldA*	*kan*, RSF *ori*, pSG5, *bldA* (*SIRAN_RS27980*)::*aac(3)IV*, *oriT,* Kan^R	This study
pKOSi_*bldH*	*kan*, RSF *ori*, pSG5, *bldH* (*SIRAN6239*), Kan^R	This study
pKOSi_Δ*bldH*	*kan*, RSF *ori*, pSG5, *bldH* (*SIRAN6239*)::*aac(3)IV*, *oriT,* Kan^R	This study

Supplementary Table 5: Primers used in this study. Homology arms for flanking regions of the genes to be deleted are depicted in bold letters. FW, forward; RV, reverse.

Name	Sequence	Description and targets
OMK_116	GCT TCA aag ctt CAT GGT CAC TTG CTC TCC TC	Cloning of *bldD* (*SIRAN2101*) and *Hin*dIII recognition sequence. FW
OMK_117	CAT TGT tct aga GGC GAT TTC CTC ACC ATC TC	Cloning of *bldD* (*SIRAN2101*) and *Xba*I recognition sequence. RV
OMK_118	**CAC AGC CGC ACG TCG ATA CAG CGT CCG GGG AGC TTT ATG** ATT CGG GGG ATC CGT CGA CC	Amplification of resistance cassette and insertion of homologous flanking regions of *bldD* (*SIRAN2101*). FW
OMK_119	**CGG CAC GTT TCT GCT GGT GAA CAG GGA AAG GGG GAC TCA** TGT AGG CTG GAG CTG CTT	Amplification of resistance cassette and insertion of homologous flanking regions of *bldD* (*SIRAN2101*). RV
OMK_148	CCT TGG CGG AAA AGT TGA TC	PCR for Southern blot probe for *bldD* (*SIRAN2101*). FW
OMK_149	CAA ATC CGA CCG TCC TTT AAG	PCR for Southern blot probe for *bldD* (*SIRAN2101*). RV
oTN681	CAT TCC tct aga GCA TGG AGA AGG TGT TGA CG	Amplification of *bldA* (*SIRAN_RS27980*) and flanking regions. *Xba*I restriction site. FW
oTN682	GAC TGT aag ctt GCG AGG AGA ACG TGT ACG TC	Amplification of *bldA* (*SIRAN_RS27980*) and flanking regions. *Hin*dIII restriction site. RV
oTN683	**GCC GTA TGC GCA CCG TAC GGC CCA GGG GGA ACC GCT GCC** ATT CCG GGG ATC CGT CGA CC	Amplification of *aac(3)IV* with overlap to the *bldA* (*SIRAN_RS27980*) flanking regions. FW
oTN684	**CCA CAC ACG CTG AGT GCG GCC ACC GGA TCT GAA GGT GCC** TGT AGG CTG GAG CTG CTT C	Amplification of *aac(3)IV* with overlap to the *bldA* (*SIRAN_RS27980*) flanking regions. RV

oTN695	CGG ACC AGC GAT CGT TGT AC	Amplification of *bldA* (*SIRAN_RS27980*) or *aac(3)IV* to check for insertion of the resistance cassette. FW
oTN696	GTG GAA TGC AGA CAC GGC GAG	Amplification of *bldA* (*SIRAN_RS27980*) to check for presence of wild-type gene. RV
oTN697	GTC GGC GAA GAC GTA GAC AT	Southern blot probe for verification of correct deletion of *bldA* (*SIRAN_RS27980*). FW
oTN698	GCG ATA AGC CTC AAG CTG AG	Southern blot probe for verification of correct deletion of *bldA* (*SIRAN_RS27980*). RV
oTN569	CAT TCC tct aga GCT TCC TGA GCG CCT TGT AC	Amplification of *bldH* (*SIRAN6239*) and flanking regions. *Xba*I restriction site. FW
oTN570	TCC AGC TCA TGG CAG ATG TC	Amplification of *bldH* (*SIRAN6239*) and flanking regions. RV
oTN571	**CCG AGA GGA CGC GAC CAC CGA GGG GGG CTT AGT GCC ATG** ATT CCG GGG ATC CGT CGA CC	Amplification of *aac(3)IV* with overlap to the *bldH* (*SIRAN6239*) flanking regions. FW
oTN572	**CGC AGT CGA TCC ACA CCA TGC GAT CGT TCA TAC GTC TCA** TGT AGG CTG GAG CTG CTT C	Amplification of *aac(3)IV* with overlap to the *bldH* (*SIRAN6239*) flanking regions. RV
oTN628	ATC TCG CAG TCG ATC CAC AC	Amplification of *bldH* (*SIRAN6239*) or *aac(3)IV* to check for insertion of the resistance cassette. FW
oTN629	CGC TAT CTG GAC AGG TCG TT	Amplification of *bldH* (*SIRAN6239*) to check for presence of wild-type gene. RV
oTN637	CGT ATG AGA GGC GAC GTC G	Southern blot probe for verification of correct deletion of *bldH* (*SIRAN6239*). FW
oTN638	GTG CTG GGC GTC GTC AAT GTC	Southern blot probe for verification of correct deletion of *bldH* (*SIRAN6239*). RV
MM025_for	AAGGCTGAAGATCATCGTGG	Amplification of *orsA* (*AN7909*) 5’ flanking region. FW
MM026_rev	GGTGACTTAAAGAGAAGATAATTAG	Amplification of *orsA* (*AN7909*) 5’ flanking region. RV
MM030_for	ATGGCTCCAAATCACGTTC	Amplification of *orsA* (*AN7909*). FW

MM031_rev	ATCACTCGGCGATAGAGAGC	Amplification of *orsA* (*AN7909*). RV
MM027_for	**AATATCTAATTATCTTCTCTTTAAGTCA**CCATGGTCTTCACACTCGAAGATTTC	Amplification of *nLuc-GFPs* fusion. FW
MM028_rev	TTACTTGTACAGCTCGTCCATG	Amplification of *nLuc-GFPs* fusion. RV
MM032_for	**GGCCCAGGGGGCTCTCTATCGCCGAGTGA**TTGCATGCCTGCAGGTCGACT	Amplification of linearized pUC18 plasmid. FW
MM033_rev	**CCCATGTGGCCCACGATGATCTTCAGCCTT**TGGCACTGGCCGTCGTTTTAC	Amplification of linearized pUC18 plasmid. RV
MM061_for	**ACTCTCGGCATGGACGAGCTGTACAAGTAA**TGCCAGATCTGTAGAAAGGTC	Amplification of *pabaA* (AN1545) gene. FW
MM062_rev	**CGGGAAAAAAAGAACGTGATTTGGAGCCAT**ATCTGGACATGCGACGGAG	Amplification of *pabaA* (AN1545) gene. RV
MM065_SB_for	AGAAGTGTGGTCTTACGATGTAC	Amplification of *orsA (AN7909)* Southern blot probe. FW
ITS1	TCCGTAGGTGAACCTGCGG	ITS region. FW
ITS4	TCCTCCGCTTATTGATATGC	ITS region. RV
orsA FW	CTATACCACCGATAGCCAGGAC	qRT-PCR primer *orsA* (*AN7909*). FW
orsA RV	CAGTGAGCAGGGCAAAGAAG	qRT-PCR primer *orsA* (*AN7909*). RV
Actin FW	CACCCTTGTTCTTGTTTTGCTC	qRT PCR primer for actin gene *acnA* (*AN6542*). FW
Actin RV	AAGTTCGCTTTGGCAACGC	qRT PCR primer for actin gene *acnA* (*AN6542*). RV

Source Data

Uncropped Southern Blots

S. iranensis* Δ*bldD

Uncropped Southern Blot verifying successful deletion of the *bldD* gene in *S. iranensis* (see Supplementary Fig. 1).

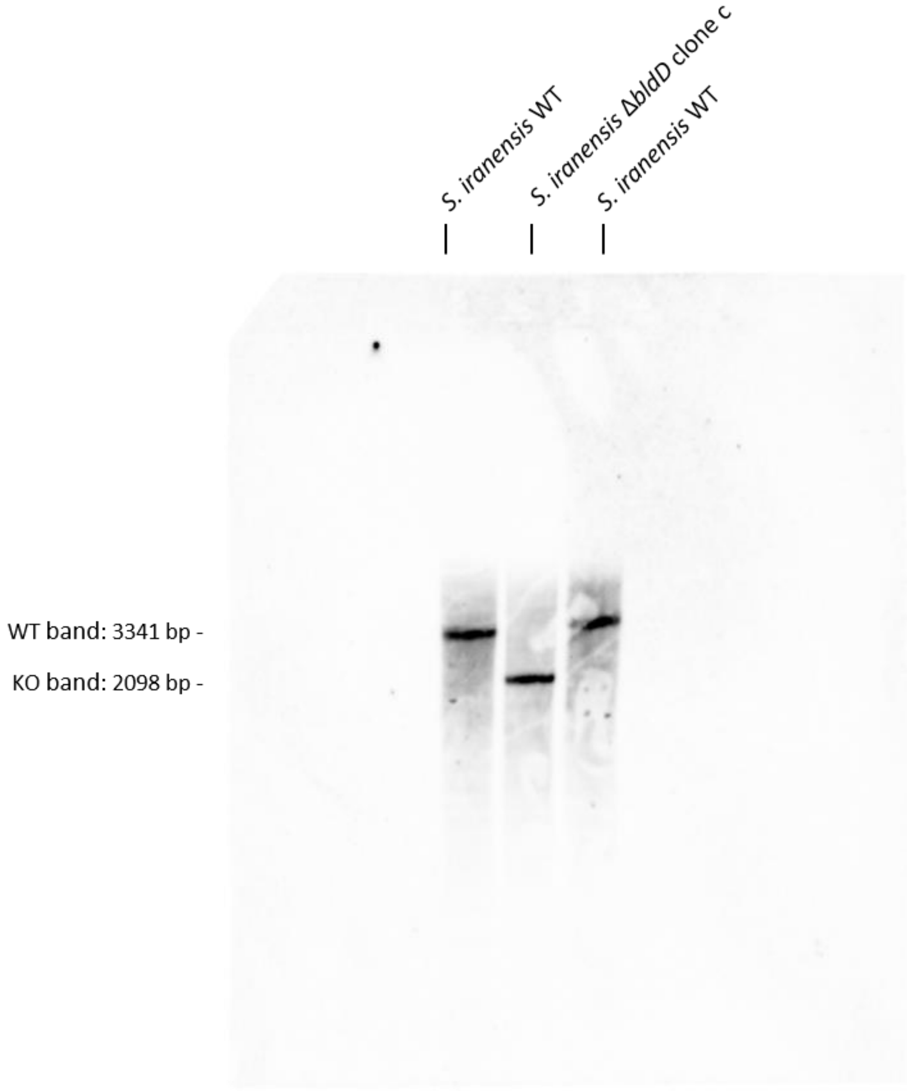

***S. iranensis* ΔbldA**

Uncropped Southern Blot verifying successful deletion of the *bldA* gene in *S. iranensis* (see Supplementary Fig. 1).

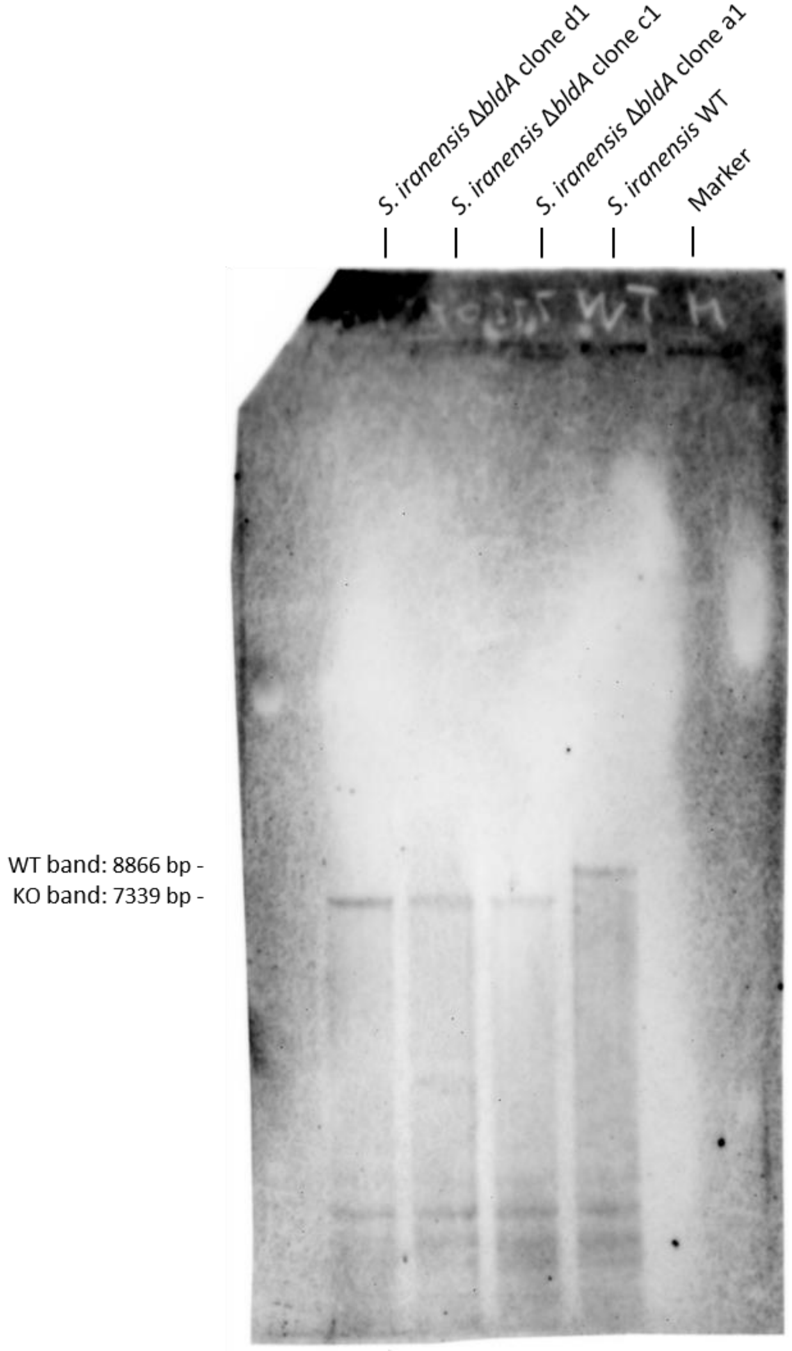

***S. iranensis* ΔbldH**

Uncropped Southern Blot verifying successful deletion of the *bldH* gene in *S. iranensis* (see Supplementary Fig. 1).

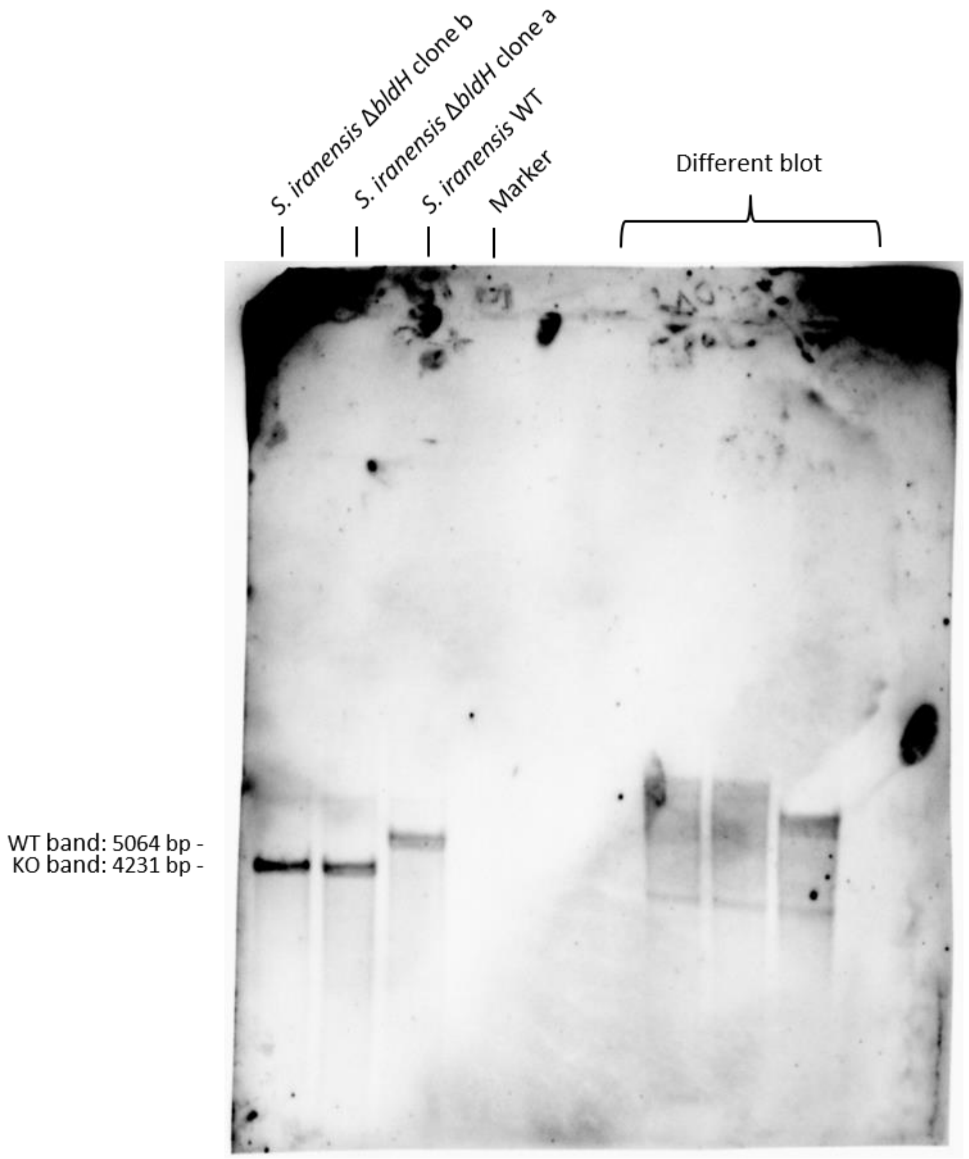

References

Supplementary Information

1 Sosio, M. *et al.* Analysis of the pseudouridimycin biosynthetic pathway provides insights into the formation of C-nucleoside antibiotics. *Cell Chem. Biol.* **25**, 540-549 (2018).

2 Hoefler, B. C., Konganti, K. & Straight, P. D. *De Novo* assembly of the *Streptomyces* sp. strain Mg1 genome using PacBio single-molecule sequencing. *Genome Announc.* **1**, e00535-00513, https://doi.org/10.1128/genomeA.00535-13 (2013).

3 Hamedi, J. *et al. Streptomyces iranensis* sp. nov., isolated from soil. *Int. J. Syst. Evol. Microbiol.* **60**, 1504-1509 (2010).

4 Krespach, M. K. C. *et al.* Lichen-like association of *Chlamydomonas reinhardtii* and *Aspergillus nidulans* protects algal cells from bacteria. *ISME J.* **14**, 2794-2805 (2020).

5 Dolak, L. *et al.* Desertomycin: Purification and physical-chemical properties. *J. Antibiot.* **36**, 13-19 (1983).

6 Stringer, M., Dean, R., Sewall, T. & Timberlake, W. Rodletless, a new *Aspergillus* developmental mutant induced by directed gene inactivation. *Genes Dev.* **5**, 1161-1171 (1991).

7 Hearn, V. M. & Mackenzie, D. W. R. Mycelial antigens from two strains of *Aspergillus fumigatus*: An analysis by two-dimensional immunoelectrophoresis. *Mycoses* **23**, 549-562 (1980).

8 d'Enfert, C. Selection of multiple disruption events in *Aspergillus fumigatus* using the orotidine-5'-decarboxylase gene, *pyrG*, as a unique transformation marker. *Curr. Genet.* **30**, 76-82 (1996).

9 Datsenko, K. A. & Wanner, B. L. One-step inactivation of chromosomal genes in *Escherichia coli* K-12 using PCR products. *Proc. Natl. Acad. Sci. U.S.A.* **97**, 6640-6645 (2000).

10 MacNeil, D. J. *et al.* Analysis of *Streptomyces avermitilis* genes required for avermectin biosynthesis utilizing a novel integration vector. *Gene* **111**, 61-68 (1992).

11 Gust, B., Challis, G. L., Fowler, K., Kieser, T. & Chater, K. F. PCR-targeted *Streptomyces* gene replacement identifies a protein domain needed for biosynthesis of the sesquiterpene soil odor geosmin. *Proc. Natl. Acad. Sci. U.S.A.* **100**, 1541-1546 (2003).

12 Paget, M. S. B., Chamberlin, L., Atrih, A., Foster, S. J. & Buttner, M. J. Evidence that the extracytoplasmic function sigma Factor ςE Is required for normal cell wall structure in *Streptomyces coelicolor* A3(2). *J. Bacteriol.* **181**, 204-211 (1999).

13 Netzker, T. *et al.* An efficient method to generate gene deletion mutants of the rapamycin-producing bacterium *Streptomyces iranensis* HM 35. *Appl. Environ. Microbiol.* **82**, 3481-3492 (2016).

Extended Data Table 2:

73 Benallaoua, S. *et al.* The mode of action of a nonpolyenic antifungal (desertomycin) produced by a strain of *Streptomyces spectabilis*. *Can. J. Microbiol.* **36**, 609-616 (1990).

74 Dolak, L. *et al.* Process for production of antibiotic U-64,767 using *Streptomyces macronensis* NRRL 12566 USA patent (1983).

75 Baldacci, E., Locci, R. & Farina, G. Studio di una nuova specie di *Streptomyces*: *Streptomyces nobilis* sp. nov. e esame di ceppi affini appartenenti ai generi *Streptomyces*, *Streptoverticillium* e *Nocardia*. *Mycopathologia et mycologia applicata* **26**, 333-348 (1965).

76 Hashimoto, T. *et al.* Identification, cloning and heterologous expression of biosynthetic gene cluster for desertomycin. *J. Antibiot.* **73**, 650-654 (2020).

77 Fan, L.-X., Guo, Z.-y. & Wu, W.-J. Isolation and characterization of *Streptomyces alboflavus* SC11 producing desertomycin A. *Afr. J. Microbiol. Res.*, 1246-1252 (2012).

78 Zeeck, A. *et al.* Oasomycine, Verfahren zu ihrer Herstellung und ihre Verwendung Germany patent (1991).

79 Shinobu, R. & Shimada, Y. On a new whirl-forming species of *Streptomyces*. *Bot. Mag. Tokyo* **75**, 170-175 (1962).

80 Sawazaki, T. *et al.* Streptomycin production by a new strain *Streptomyces mashuensis*. *J. Antibiot.* **8**, 44-47 (1955).

81 Kuo, M.-S. *et al.* Monazomycin B, a new macrolide antibiotic of the monazomycin family. *J. Antibiot.* **43**, 438-440 (1990).

82 Hamedi, J. *et al.* *Streptomyces iranensis* sp. nov., isolated from soil. *Int. J. Syst. Evol. Microbiol.* **60**, 1504-1509 (2010).

83 Kumar, Y. & Goodfellow, M. Five new members of the *Streptomyces violaceusniger* 16S rRNA gene clade: *Streptomyces castelarensis* sp. nov., comb. nov., *Streptomyces himastatinicus* sp. nov., *Streptomyces mordarskii* sp. nov., *Streptomyces rapamycinicus* sp. nov. and *Streptomyces ruanii* sp. nov. *Int. J. Syst. Evol. Microbiol.* **58**, 1369-1378 (2008).

84 Cheng, J. *et al.* Azalomycin F complex is an antifungal substance produced by *Streptomyces malaysiensis* MJM1968 isolated from agricultural soil. *J. Korean Soc. Appl. Biol. Chem.* **53**, 545-552 (2010).

85 Yuan, G., Hong, K., Lin, H., She, Z. & Li, J. New azalomycin F analogs from mangrove *Streptomyces* sp. 211726 with activity against microbes and cancer cells. *Mar. Drugs* **11**, 817-829 (2013).

86 Höltzl, A. *et al.* Spirofungin, a new antifungal antibiotic from *Streptomyces violaceusniger* Tü 4113. *J. Antibiot.* **51**, 699-707 (1998).

87 Kim, K. H. *et al.* Natalamycin A, an ansamycin from a termite-associated *Streptomyces* sp. *Chem. Sci.* **5**, 4333-4338 (2014).

88 Arcamone, F. M., Bertazzoli, C., Ghione, M. & Scotti, T. Melanosporin and elaiophylin, new antibiotics from *Streptomyces melanosporus* (sive *melonsporofaciens*) n. sp. *Giornale di Microbiologia* **7**, 207-216 (1959).

89 Sembiring, L., Ward, A. C. & Goodfellow, M. Selective isolation and characterisation of members of the *Streptomyces violaceusniger* clade associated with the roots of *Paraserianthes falcataria*. *Antonie van Leeuwenhoek* **78**, 353-366 (2000).

90 Kusuma, A. B., Nouioui, I. & Goodfellow, M. Genome-based classification of the *Streptomyces violaceusniger* clade and description of *Streptomyces sabulosicollis* sp. nov. from an Indonesian sand dune. *Antonie van Leeuwenhoek* **114**, 859-873 (2021).

91 Nammali, A. *et al. Streptomyces endocoffeicus* sp. nov., an endophytic actinomycete isolated from *Coffea arabica* (L.). *Antonie van Leeuwenhoek* **114**, 1889-1898 (2021).

92 Di, R., Low, Y. C., Wang, L., Luo, Y. & Cuomo, C. A. Draft genome sequence of *Streptomyces aureoverticillatus* HN6, a strain antagonistic against *Fusarium oxysporum* f. sp. *cubense* race 4. *Microbiol. Resour. Announc.* **9**, e00210-00220, https://doi.org/10.1128/MRA.00210-20 (2020).

93 Hamm, P. S. *et al. Streptomyces buecherae* sp. nov., an actinomycete isolated from multiple bat species. *Antonie van Leeuwenhoek* **113**, 2213-2221 (2020).

94 Zhou, S., Yang, X., Huang, D. & Huang, X. *Streptomyces solisilvae* sp. nov., isolated from tropical forest soil. *Int. J. Syst. Evol. Microbiol.* **67**, 3553-3558 (2017).

95 Fyans, J. K., Bown, L. & Bignell, D. R. D. Isolation and characterization of plant-pathogenic *Streptomyces* species associated with common scab-infected potato tubers in Newfoundland. *Phytopathology* **106**, 123-131 (2016).

96 Saintpierre, D., Amir, H., Pineau, R., Sembiring, L. & Goodfellow, M. *Streptomyces yatensis* sp. nov., a novel bioactive streptomycete isolated from a New-Caledonian ultramafic soil. *Antonie van Leeuwenhoek* **83**, 21-26 (2003).

97 Blodgett, J. A. V. *et al.* Common biosynthetic origins for polycyclic tetramate macrolactams from phylogenetically diverse bacteria. *Proc. Natl. Acad. Sci. U.S.A.* **107**, 11692-11697 (2010).

98 Park, J.-S. *et al.* Genome Analysis of *Streptomyces nojiriensis* JCM 3382 and distribution of gene clusters for three antibiotics and an azasugar across the genus

Streptomyces. *Microorganisms* **9**, 1802, https://doi.org/10.3390/microorganisms9091802 (2021).

99 *Bergey's Manual of Systematic Bacteriology*. 2 edn, (Springer, New York, 2012).

100 Guerrero-Garzón, J. F. *et al. Streptomyces* spp. from the marine sponge *Antho dichotoma*: Analyses of secondary metabolite biosynthesis gene clusters and some of their products. *Front. Microbiol.* **11**, 437, https://doi.org/10.3389/fmicb.2020.00437 (2020).

101 Hayakawa, Y., Kanamaru, N., Shimazu, A. & Seto, H. Lydicamycin, a new antibiotic of a novel skeletal type I. Taxonomy, fermentation, isolation and biological activity. *J. Antibiot.* **44**, 282-287 (1991).

102 Komaki, H., Ichikawa, N., Hosoyama, A., Fujita, N. & Igarashi, Y. Draft genome sequence of marine-derived *Streptomyces* sp. TP-A0598, a producer of anti-MRSA antibiotic lydicamycins. *Stand. Genom. Sci.* **10**, 58, https://doi.org/10.1186/s40793-015-0046-5 (2015).

103 Komaki, H., Hosoyama, A., Igarashi, Y. & Tamura, T. *Streptomyces lydicamycinicus* sp. nov. and its secondary metabolite biosynthetic gene clusters for polyketide and nonribosomal peptide compounds. *Microorganisms* **8**, 370, https://doi.org/10.3390/microorganisms8030370 (2020).

104 Tistechok, S. I. *et al.* Genetic identification and antimicrobial activity of *Streptomyces* sp. strain Je 1–6 isolated from rhizosphere soil of *Juniperus excelsa* Bieb. *Cytol. Genet.* **55**, 28-35 (2021).

105 Liu, D. *et al.* Antifungal, plant growth-promoting, and genomic properties of an endophytic actinobacterium *Streptomyces* sp. NEAU-S7GS2. *Front. Microbiol.* **10**, 2077, https://doi.org/10.3389/fmicb.2019.02077 (2019).

106 Furumai, T. *et al.* TPU-0037-A, B, C and D, novel lydicamycin congeners with anti-MRSA activity from *Streptomyces platensis* TP-A0598. *J. Antibiot.* **55**, 873-880 (2002).
