# Supplementary material for: Streptomyces polyketides mediate bacteria–fungi interactions across soil environments
Source: Nat Microbiol. 2023 Jun 15;8(7):1348–61. doi: 10.1038/s41564-023-01382-2 (PMC10322714; doi:10.1038/s41564-023-01382-2)
Supplement: Supplementary file 2 — Reporting Summary [file 41564_2023_1382_MOESM2_ESM.pdf]

nature portfolio

Corresponding author(s): Axel A. Brakhage
Last updated by author(s): Apr 7, 2023

Reporting Summary

Nature Portfolio wishes to improve the reproducibility of the work that we publish. This form provides structure for consistency and transparency in reporting. For further information on Nature Portfolio policies, see our Editorial Policies and the Editorial Policy Checklist.

Statistics

For all statistical analyses, confirm that the following items are present in the figure legend, table legend, main text, or Methods section.

n/a	Confirmed	
☐	☒	The exact sample size (*n*) for each experimental group/condition, given as a discrete number and unit of measurement
☒	☐	A statement on whether measurements were taken from distinct samples or whether the same sample was measured repeatedly
☐	☒	The statistical test(s) used AND whether they are one- or two-sided<br>*Only common tests should be described solely by name; describe more complex techniques in the Methods section.*
☒	☐	A description of all covariates tested
☒	☐	A description of any assumptions or corrections, such as tests of normality and adjustment for multiple comparisons
☐	☒	A full description of the statistical parameters including central tendency (e.g. means) or other basic estimates (e.g. regression coefficient) AND variation (e.g. standard deviation) or associated estimates of uncertainty (e.g. confidence intervals)
☐	☒	For null hypothesis testing, the test statistic (e.g. *F*, *t*, *r*) with confidence intervals, effect sizes, degrees of freedom and *P* value noted<br>*Give P values as exact values whenever suitable.*
☒	☐	For Bayesian analysis, information on the choice of priors and Markov chain Monte Carlo settings
☒	☐	For hierarchical and complex designs, identification of the appropriate level for tests and full reporting of outcomes
☒	☐	Estimates of effect sizes (e.g. Cohen's *d*, Pearson's *r*), indicating how they were calculated

Our web collection on statistics for biologists contains articles on many of the points above.

Software and code

Policy information about availability of computer code

Data collection	Fluorescence pictures were acquired on a Keyence BZ-X800 microscope using BZ-X800 Viewer. Luminescence measurements were made in a Tecan microtiter plate reader using the Tecan i-control (version 3.1.9.0).
Data analysis	The mass spectrometry data was searched against the NCBI database of Streptomyces iranensis (2019/01/24) using the Proteome Discoverer 2.2 (Thermo Fisher Scientific) and the algorithms of Sequest HT (version of PD2.2), Mascot 2.4 and MS Amanda 2.0. RNA-Seq reads were aligned to the NCBI reference genome for S. iranensis (Assembly GCA_000938975.1). qPCR data were obtained using the instruments´ software QuantStudio Design & Analysis Software v1.5.2. LC-MS data were analyzed using Xcalibur software v4.4.16.14. Data was processed using Excel and Powerpoint (Microsoft Office Standard 2019), Graphpad 9 and Inkscape (version 1.2). Chemical structures were created using ChemDraw (version 20.1.1). Analysis of microscopic pictures was done using BZ-X800 analyzer (version 1.1.1.8). Potential natural product (NP) biosynthesis gene clusters (BGCs) were identified using antiSMASH and the BLAST algorithm. Whole genome-based phylogenetic analysis was carried out using the Type Strain Genome Server (TYGS). Obtained ITS sequences were analyzed using the BLAST algorithm and MEGA X. MALDI Spectra were processed in flexAnalysis 3.3, uploaded in flexImaging 3.0 for visualization and SCILS Lab 2015b for analysis and representation.

For manuscripts utilizing custom algorithms or software that are central to the research but not yet described in published literature, software must be made available to editors and reviewers. We strongly encourage code deposition in a community repository (e.g. GitHub). See the Nature Portfolio guidelines for submitting code & software for further information.

Data

Policy information about availability of data

All manuscripts must include a data availability statement. This statement should provide the following information, where applicable:

- Accession codes, unique identifiers, or web links for publicly available datasets
- A description of any restrictions on data availability
- For clinical datasets or third party data, please ensure that the statement adheres to our policy

We have the following statement in the manuscript: "The authors declare that all data supporting the findings of this study are available within this article, its supplementary information, source data or in public repositories.
RNAseq data: Gene Expression Omnibus (GEO), accession number GSE201630 (https://www.ncbi.nlm.nih.gov/geo/query/acc.cgi?acc=GSE201630).
DNAseq data: NCBI BioProject PRJNA830323 (https://www.ncbi.nlm.nih.gov/bioproject/PRJNA830323).
Mass spectrometry proteomics data: PRIDE, dataset identifier PXD033242 (https://www.ebi.ac.uk/pride/archive/projects/PXD033242).
ITS sequences: NCBI accession numbers ON307333 – ON307363 (https://www.ncbi.nlm.nih.gov/nuccore/?term=ON307333:ON307363%5Baccn%5D)."

Human research participants

Policy information about studies involving human research participants and Sex and Gender in Research.

Reporting on sex and gender	N/A
Population characteristics	N/A
Recruitment	N/A
Ethics oversight	N/A

Note that full information on the approval of the study protocol must also be provided in the manuscript.

Field-specific reporting

Please select the one below that is the best fit for your research. If you are not sure, read the appropriate sections before making your selection.

☒ Life sciences ☐ Behavioural & social sciences ☐ Ecological, evolutionary & environmental sciences

For a reference copy of the document with all sections, see nature.com/documents/nr-reporting-summary-flat.pdf

Life sciences study design

All studies must disclose on these points even when the disclosure is negative.

Sample size	No sample size calculator was used. All sample sizes were chosen as n >= 3, in order to perform the statistical analyses specified in the respective figure legends.
Data exclusions	No data were excluded, results from all samples are shown.
Replication	For qRT-PCR, 2 independent experiments were performed, each in 5-6 biological replicates. All other experiments were performed independently at least three times in >=3 biological replicates. Positive and negative controls were included in all experiments. All attempts at replication were successful.
Randomization	Randomization was not relevant for the study as we did not perform group comparisons.
Blinding	No blinding was performed in our study as no group comparisons were performed. qPCR data were obtained using the instruments´software, regardless of sample type.

Reporting for specific materials, systems and methods

We require information from authors about some types of materials, experimental systems and methods used in many studies. Here, indicate whether each material, system or method listed is relevant to your study. If you are not sure if a list item applies to your research, read the appropriate section before selecting a response.

Materials & experimental systems

n/a	Involved in the study
☒	☐ Antibodies
☒	☐ Eukaryotic cell lines
☒	☐ Palaeontology and archaeology
☒	☐ Animals and other organisms
☒	☐ Clinical data
☒	☐ Dual use research of concern

Methods

n/a	Involved in the study
☒	☐ ChIP-seq
☒	☐ Flow cytometry
☒	☐ MRI-based neuroimaging
