# Supplementary material for: Streptomyces polyketides mediate bacteria–fungi interactions across soil environments
Source: Nat Microbiol. 2023 Jun 15;8(7):1348–61. doi: 10.1038/s41564-023-01382-2 (PMC10322714; doi:10.1038/s41564-023-01382-2)

A. nidulans orsAp-nLuc-GFPs

Uncropped Southern Blot verifying successful insertion of the *nLuc-GFPs* translational gene fusion in the *orsA* genomic locus of *A. nidulans* (see Extended Data Fig. 6b).

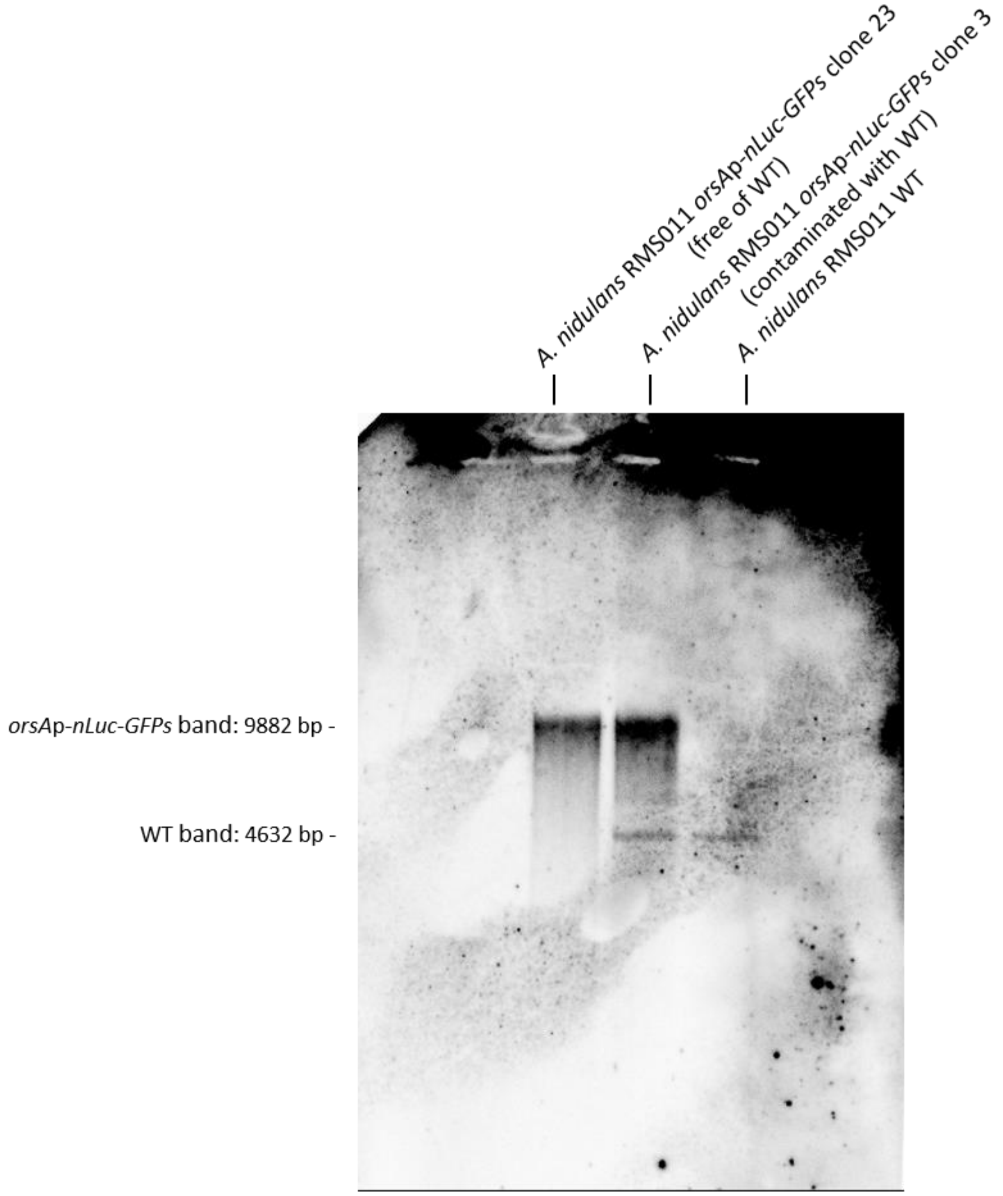

Supplement: Supplementary file 4 — Unprocessed Southern blot. [file 41564_2023_1382_MOESM4_ESM.pdf]
